# Therapeutic In Vivo Gene Editing Achieved by a Hypercompact CRISPR‐Cas12f1 System Delivered with All‐in‐One Adeno‐Associated Virus

**DOI:** 10.1002/advs.202308095

**Published:** 2024-02-26

**Authors:** Tongtong Cui, Bingyu Cai, Yao Tian, Xin Liu, Chen Liang, Qingqin Gao, Bojin Li, Yali Ding, Rongqi Li, Qi Zhou, Wei Li, Fei Teng

**Affiliations:** ^1^ Key Laboratory of Organ Regeneration and Reconstruction, State Key Laboratory of Stem Cell and Reproductive Biology Institute of Zoology Chinese Academy of Sciences Beijing 100101 China; ^2^ Institute for Stem Cell and Regeneration Chinese Academy of Sciences Beijing 100101 China; ^3^ University of Chinese Academy of Sciences Beijing 101408 China; ^4^ Beijing Institute for Stem Cell and Regenerative Medicine Beijing 100101 China

**Keywords:** AAV delivery, CRISPR‐Cas12f1, gene editing, gene therapy, retinitis pigmentosa

## Abstract

CRISPR‐based gene therapies are making remarkable strides toward the clinic. But the large size of most widely used Cas endonucleases including Cas9 and Cas12a restricts their efficient delivery by the adeno‐associated virus (AAV) for in vivo gene editing. Being exceptionally small, the recently engineered type V‐F CRISPR‐Cas12f1 systems can overcome the cargo packaging bottleneck and present as strong candidates for therapeutic applications. In this study, the pairwise editing efficiencies of different engineered Cas12f1/sgRNA scaffold combinations are systemically screened and optimized, and the CasMINI_v3.1/ge4.1 system is identified as being able to significantly boost the gene editing activity. Moreover, packaged into single AAV vectors and delivered via subretinal injection, CasMINI_v3.1/ge4.1 achieves remarkably high in vivo editing efficiencies, over 70% in transduced retinal cells. Further, the efficacy of this Cas12f1 system‐based gene therapy to treat retinitis pigmentosa in *Rho* ^P23H^ mice is demonstrated by the therapeutic benefits achieved including rescued visual function and structural preservation. And minimal bystander editing activity is detected. This work advances and expands the therapeutic potential of the miniature Cas12f1 system to support efficient and accurate in vivo gene therapy.

## Graphical abstract

A hypercompact mini‐CRISPR system suitable for all‐in‐one AAV delivery, CasMINI_v3.1/ge4.1, is identified to achieve highly efficient and accurate in vivo gene editing. This Cas12f1‐based system is employed to cure retinal degeneration in a mouse model of genetic blindness in this study, suggesting its promising applications for in vivo gene therapy.

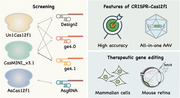

## 1 Introduction

The advent of precise and facile genome engineering using the bacterial RNA‐guided CRISPR‐Cas9 system has revolutionized our ability to edit genetic information. Due to its high specificity, efficiency, versatility, and simplicity, CRISPR technology was soon widely adopted and rapidly applied in the biomedical and therapeutic space.^[^ ^1^, ^2^ ^]^ To cope with the varied and complex application scenarios, a variety of efficient Cas variants and derivatives have been developed and harnessed for mammalian genome editing.^[^ ^3^ ^]^ Among them, Cas9 and Cas12a nucleases are the most prevalent and have already been employed in gene therapy to treat genetic diseases and cancer.^[^ ^4^, ^5^ ^]^

Gene therapy aims to treat and cure human diseases via the transfer of genetic materials to achieve gene supplementation, gene silencing, or precise modifications of the cellular genome.^[^ ^6^ ^]^ Depending on the accessibility of the target cells, gene therapy can be performed ex vivo or in vivo.^[^ ^7^ ^]^ To treat certain diseases like hemoglobinopathies and cancer, the patient‐derived hematopoietic stem cells and immune cells can be genetically engineered ex vivo, respectively.^[^ ^7^ ^]^ However, most inherited genetic diseases and metabolic disorders call for an efficient in vivo delivery of the genetic agents into target tissues or cells directly.^[^ ^7^ ^]^ To achieve this goal, virus‐ and non‐virus‐based vehicles have been developed and substantially improved.^[^ ^8^ ^]^ Among them, the AAV vector, as a US Food and Drug Administration (FDA)‐approved vehicle, has been the leading delivery platform for in vivo gene therapy, owing to its broad tropism, low immunogenicity, long‐term expression, and ease of production.^[^ ^9^ ^]^ Nevertheless, a major drawback of AAV vectors is their limited packaging capacity (≈4.7 kb), which hinders the delivery of large genes.

CRISPR technology‐based gene editing has opened up new possibilities to address a broad spectrum of genetic diseases and is now moving rapidly into the clinic.^[^ ^10^ ^]^ However, the most widely‐used RNA‐guided Cas effectors including Cas9 and Cas12a are relatively large (950–1400 amino acids),^[^ ^4^ ^]^ which significantly restricts their delivery into target tissues by the cargo‐size‐limited AAV platform. Driven by the strong interest and demand from both academia and industry, new miniature CRISPR/Cas platforms are emerging. The recently defined Cas12f effectors are exceptionally hypercompact in size (400–700 amino acids), which overcomes the load limitation of AAV vectors and makes them packageable into a single AAV particle.^[^ ^11^, ^12^, ^13^, ^14^ ^]^

Classified as a class 2 type V‐F CRISPR endonuclease, the hypercompact Cas12f1 (also known as Cas14a1), was initially identified from archaea with minimal editing activity in mammalian cells.^[^ ^15^, ^16^ ^]^ In the following studies, besides defining novel active Cas12f1 orthologs, the original non‐functional Cas12f1 formats had been transformed into highly efficient genome editors through extensive engineering of both the Cas12f1 effector and single guide RNA (sgRNA), which were also benefiting from relevant structural elucidation.^[^ ^11^, ^12^, ^14^, ^17^, ^18^, ^19^, ^20^, ^21^ ^]^ Particularly, the engineered Cas12f1 systems with enhanced targeting capabilities were proved to achieve genome editing in mammalian cells both in vitro and in vivo.^[^ ^11^, ^12^, ^13^, ^22^ ^]^ Despite these advances, to maximize their utility in gene therapy, the editing efficacy of CRISPR‐Cas12f1 platforms requires to be further optimized. Meanwhile, more evidence to test their therapeutic effects via an efficient in vivo delivery is remarkably important to further expand their application scenarios.

In the present work, we systematically screened and evaluated the pairwise editing efficiencies of different engineered Cas12f1/sgRNA combinations. On the basis of relative targeting efficiencies in mouse cells, CasMINI_v3.1/ge4.1 was selected as the lead candidate pair. We further tested the in vivo activity of this system by subretinal injection of the AAV vectors packaging Cas12MINI_v3.1 and sgRNA_ge4.1. Targeting the *Nr2e3* gene in photoreceptor cells of a mouse model with retinitis pigmentosa (RP), a type of most common inherited retinal disorders, we observed significantly high in vivo editing efficiencies. Notably, our results further demonstrate that efficient *Nr2e3* deletion can reprogram the *Rho* ^P23H^ mutation‐sensitive rod photoreceptors to functional cone‐like cells. This therapeutic cellular reprogramming process enables improved photoreceptor survival and partially restored visual function in *Rho* ^P23H^ mice. Moreover, using targeted deep sequencing and whole genome sequencing (WGS), we were unable to detect off‐target cleavage, suggesting the high specificity of CasMINI_v3.1/ge4.1 system. Taken together, our current results prove that the miniature Cas12f1 system is a promising gene editing tool for retinal gene therapy and additional CRISPR‐based medicines for other inherited diseases.

## 2 Results

### 2.1 CasMINI_v3.1 with sgRNA ge4.1 Outperforms Other Combinations in Targeted Editing Efficiency

To select out the pairwise combination of Cas12f1 effector and sgRNA scaffold for our proof‐of‐concept trial of in vivo gene therapy, we started with synthesizing sequences encoding Un1Cas12f1,^[^ ^11^ ^]^ CasMINI_v3.1 (engineered from Un1Cas12f1: D143R/T147R/E151A),^[^ ^17^ ^]^ and *Acidibacillus sulfuroxidans* Cas12f1 (AsCas12f1) nucleases (Supplementary Sequences, Supporting Information),^[^ ^12^ ^]^ as previously reported. Meanwhile, we also synthesized optimized sgRNA scaffolds for Un1Cas12f1 including version ge4.0, ge4.1^[^ ^11^ ^]^ and Design2,^[^ ^17^ ^]^ and AsCas12f1 sgRNA (AsgRNA) (Supplementary Sequences, Supporting Information).^[^ ^12^ ^]^ As all synthesized Cas12f1 effectors recognize 5′ T‐rich protospacer adjacent motifs (PAMs),^[^ ^16^ ^]^ we could directly compare their performance combined with different sgRNA scaffolds side by side targeting the same genomic sequences. To do this, we designed eight spacer sequences (Table S1, Supporting Information) and cloned them into the above‐mentioned different sgRNA scaffolds for Cas12f1 systems to target the mouse *Nr2e3* locus (Figure S1a, Supporting Information).

**Figure 1:**
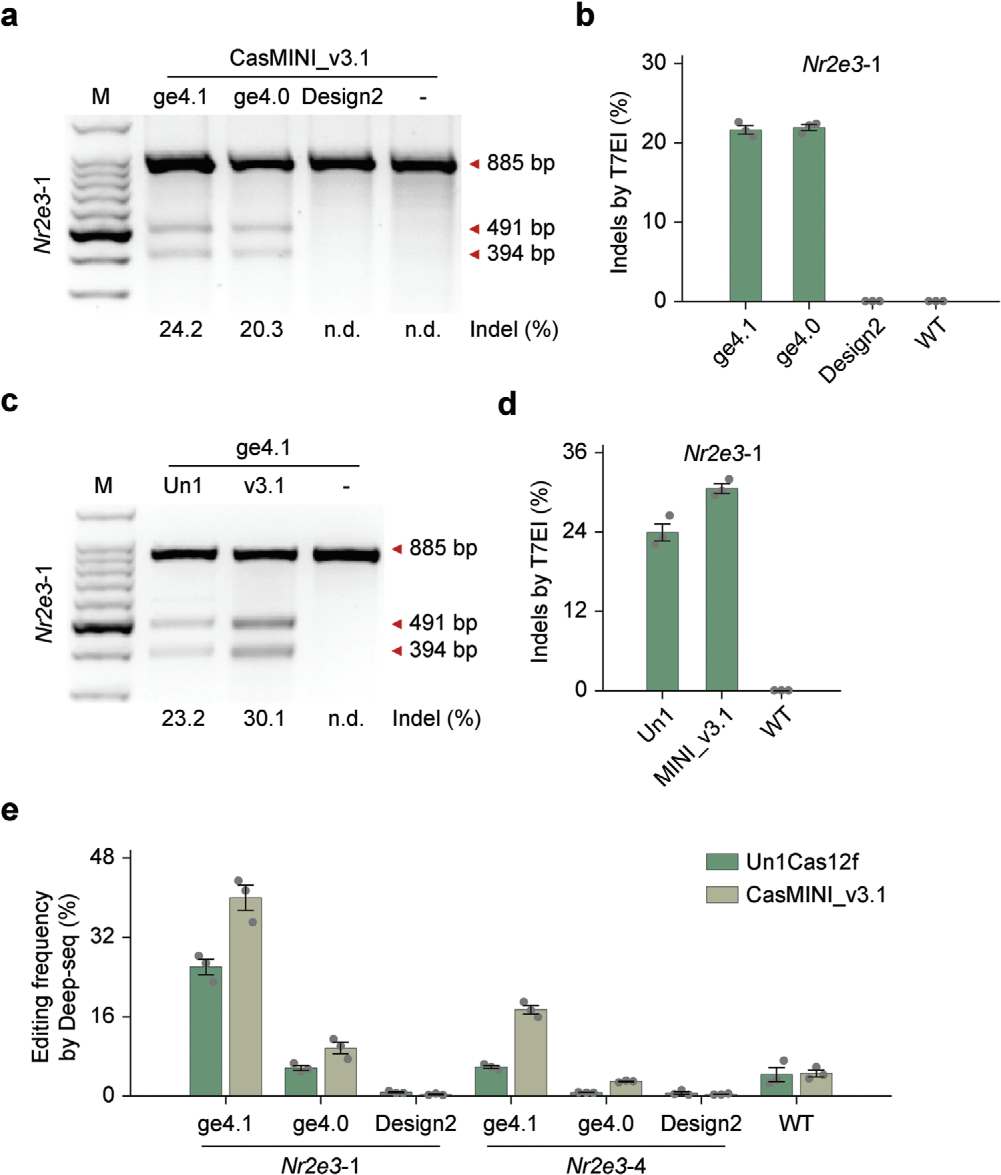
Robust genome editing using CRISPR‐Cas12f1 systems. a) T7EI analysis of the indels generated by CasMINI_v3.1 with different sgRNA scaffolds (ge4.1, ge4.0, Design2) on the mouse *Nr2e3* target site 1. The indel rate is shown under the lanes with mutation. ‐, U6 empty vector without gRNA expression. b) Indel efficiency comparison of different gRNA scaffolds on the mouse *Nr2e3* target site 1 in mouse N2A cells, determined by T7EI assay. Error bars indicate standard errors of the mean (s.e.m.), *n* = 3. c) T7EI analysis of the indels generated by Un1Cas12f1 and CasMINI_v3.1 with gRNA scaffolds ge4.1 on the mouse *Nr2e3* target site 1. The indel rate is shown under the lanes with mutation. ‐, a pCAG‐2AeGFP empty vector without Cas12f1 expression. d) Indel efficiency comparison between Un1Cas12f1 and CasMINI_v3.1 on the mouse *Nr2e3* target site 1 in mouse N2A cells, determined by T7EI assay. Error bars indicate the s.e.m., *n* = 3. e) Indel efficiency comparison of Cas12f1 (Un1Cas12f1 and CasMINI_v3.1) with different gRNA scaffolds (ge4.1, ge4.0, Design2) on the mouse *Nr2e3* target site 1 and site 4 in mouse N2A cells, determined by targeted deep sequencing. Error bars indicate the s.e.m., *n* = 3.

On‐target editing efficiencies were evaluated in mouse neuroblastoma N2A cells and indel (insertion and deletion) frequencies were calculated by T7 endonuclease I (T7EI) assay (Figure S1b,c, Supporting Information) and Sanger sequencing (Figure S1d, Supporting Information). We first identified that target sites 1 and 4 of the eight sites could be efficiently edited by the Un1Cas12f1 system (Figure S1b,d, Supporting Information). The initial testing results showed that AsCas12f1/AsgRNA^[^ ^12^ ^]^ failed to generate detectable activity at any selected sites (Figure S1c, Supporting Information). So, we excluded this editing system in the following experiments. We next compared the targeting efficiencies of three engineered sgRNA scaffolds when combined with CasMINI_v3.1. Our results showed that scaffold version ge4.1 achieves a higher efficiency than the other two scaffolds (version ge4.0 and Design2) when targeting both site 1 and site 4 of *Nr2e3* locus (**Figure** **1**a,b; Figure S2a,b, Supporting Information). We further went on to comparatively characterize the pairwise effectiveness of two Cas12f1 effectors (Un1Cas12f1_wt and CasMINI_v3.1) combined with sgRNA scaffold ge4.1 to target *Nr2e3* site 1 and site 4. The T7EI analyses showed that CasMINI_v3.1 performs better than Un1Cas12f1 (Figure 1c,d; Figure S2c,d, Supporting Information), which is consistent with the previous report as CasMINI_v3.1 was yielded by iterative protein engineering from the wildtype Un1Cas12f1.^[^ ^22^ ^]^ Hence, by sequential rounds of side‐by‐side comparative analyses of the effectiveness achieved by different combinations of Cas12f1 effectors and sgRNA scaffolds, we identified the combination of CasMINI_v3.1 and ge4.1 could achieve the most efficient editing activity at *Nr2e3* site 1 and site 4 in our tests (Figure 1e; Figure S2e, Supporting Information).

### 2.2 Efficient In Vivo Gene Editing in the Mouse Retina by All‐in‐One AAV Delivery of CasMINI_v3.1/ge4.1

**Figure 2:**
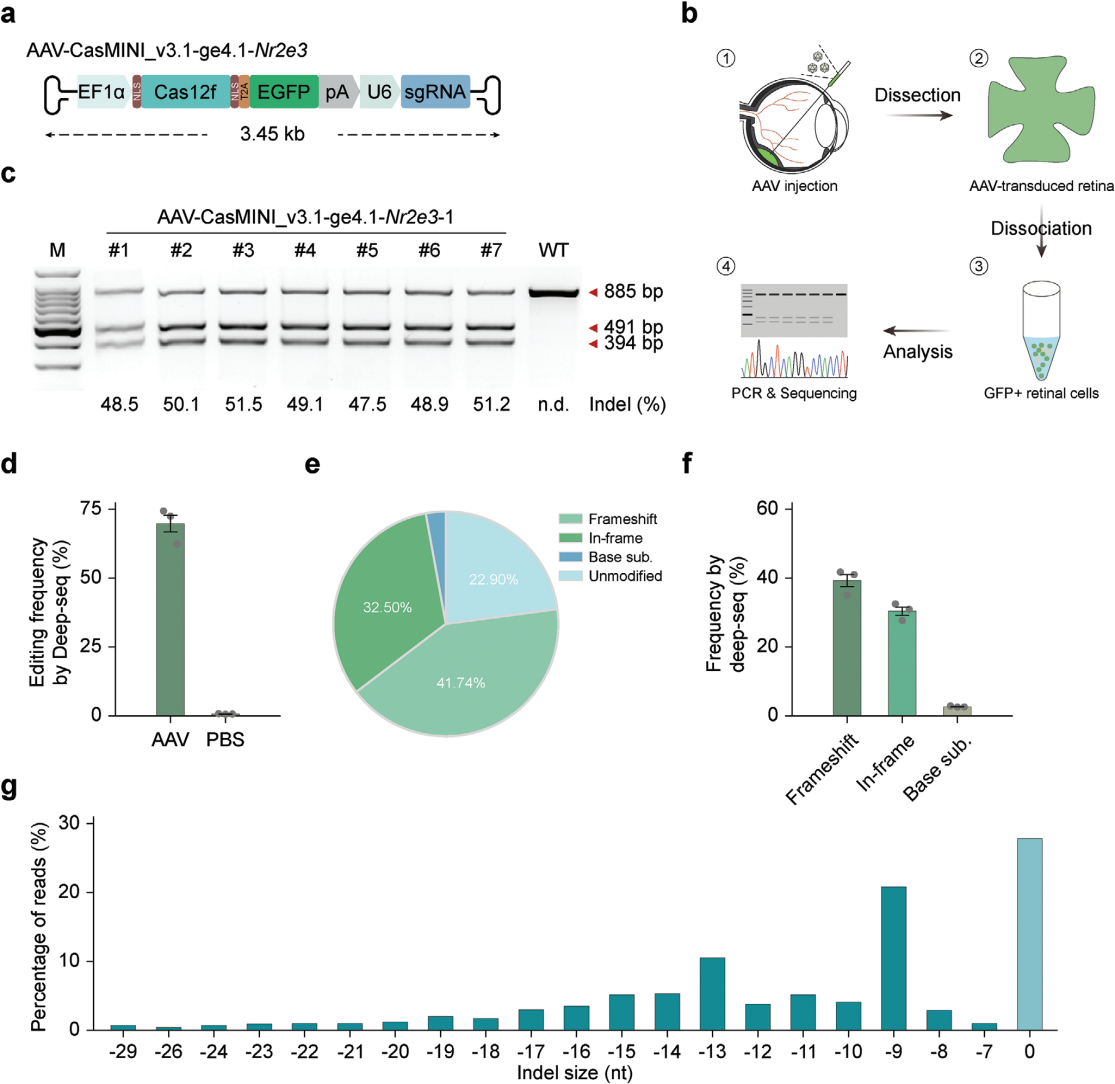
CasMINI_v3.1/ge4.1‐mediated in vivo genome editing. a) Schematic of AAV construct encoding CasMINI_v3.1 and ge4.1 for in vivo gene editing. b) A schematic illustration of AAV‐mediated in vivo genome editing and editing efficiency assessment in mouse retina. c) T7E1 analysis of the indels generated by AAV‐CasMINI_v3.1/ge4.1 on the mouse *Nr2e3* target site 1 in 7 mouse retinas. The indel rate is shown under the lanes with mutation. WT, wide‐type mouse retina injected with AAV‐GFP. d) Targeted deep sequencing analysis of editing efficiencies on the mouse *Nr2e3* target site 1 in mouse retinas using AAV‐CasMINI_v3.1/ge4.1 (indicated as AAV). PBS, negative control with PBS injection. Error bars indicate the standard error of the mean (s.e.m.), *n* = 3. e) Representative types of editing outcomes in vivo on the mouse *Nr2e3* target site 1 by CasMINI_v3.1/ge4.1. f) Average frequency of in vivo editing outcomes generated by CasMINI_v3.1/ge4.1 on the mouse *Nr2e3* target site 1. Error bars indicate the s.e.m., *n* = 3. g) Frequency distribution of alleles with indels and without indels. In this example, the indels are dominated by small deletions.

Given that CasMINI_v3.1/ge4.1 system exhibits the most robust on‐target editing efficiencies in vitro, we next sought to assess their capability to edit the *Nr2e3* gene in vivo. The hypercompact size of Cas12f1 protein makes it the best fit for in vivo delivery using a single AAV vector. Here, to deliver the CasMINI_v3.1/ge4.1 system to the subretinal space, we used recombinant AAV serotype 8 (AAV8), which displays preferential tropism for the retinal tissues.^[^ ^23^ ^]^ We first constructed CasMINI_v3.1 together with ge4.1, driven by EF1α core promoter and U6 promoter, respectively, into AAV8 vectors to target the mouse *Nr2e3* gene site 1 or site 4 (**Figure** **2**a). Notably, as a miniature CRISPR system, additional elements are allowed for the package in a single AAV particle except for the core editing system. Therefore, we additionally inserted an enhanced green fluorescent protein (EGFP)^[^ ^24^ ^]^ after CasMINI_v3.1 connected by a T2A self‐cleaving peptide^[^ ^25^ ^]^ (Figure 2a). This approach is particularly useful to facilitate the tracing as well as isolation of AAV‐transduced cells in vivo.

We set out to validate whether the AAV plasmid construction is successful before AAV production. After transfecting AAV8‐CasMINI_v3.1‐ge4.1‐*Nr2e3* plasmids into N2A cells (Figure S3a, Supporting Information), we detected comparative and robust cleavage at both sites of *Nr2e3*, respectively (Figure S3b,c, Supporting Information), indicating the effectiveness of the AAV construct. Next, we investigated the in vivo gene‐editing performance of this AAV‐Cas12f1 vector. The resulting virus particles were administered via subretinal injection into the subretinal space of wildtype C57BL/6 mice at postnatal day 14 (P14). At 1 month post injection (P45), mice were sacrificed to examine the editing efficiencies. Retinas were manually dissected and enzymatically dissociated into single cells by the Papain dissociation system. Following that, GFP‐positive (GFP^+^) cells were sorted by fluorescence‐activated cell sorting (FACS) and underwent subsequent analyses (Figure 2b). The transduction rate could reach over 70% as indicated by the ratio of GFP^+^ cells (Figure S3d, Supporting Information). Encouragingly, the T7EI assay results suggested high levels of targeted mutagenesis in the retinas (Figure 2c). More importantly, Sanger sequencing profiles generated by three independent retina samples demonstrate mutagenesis frequencies of over 80% (Figure S3e, Supporting Information). Notably, these findings were further supported by targeted‐deep sequencing analysis (Figure 2d). Moreover, by further profiling the editing outcomes, we found that the vast majority of the editing events were deletions, including frameshifts (41.74%) and in‐frame deletions (32.50%) (Figure 2e,f). In this context, in vivo CasMINI_v3.1‐edited products were highly enriched with small deletions (<30 bp) (Figure 2g). Together, these results collectively demonstrate that the all‐in‐one AAV delivery of the CasMINI_v3.1/ge4.1 system can achieve high frequencies of on‐target mutagenesis in vivo.

### 2.3 Therapeutic Gene Editing to Treat RP via CasMINI_v3.1/ge4.1‐*Nr2e3*

To further explore the utility of CasMINI_v3.1/ge4.1 in vivo, we investigated its ability to mediate therapeutic gene editing for the treatment of RP. Inherited retinal dystrophies have become the leading cause of untreatable blindness, of which RP is the most common and associated with genetic defects in over 60 genes.^[^ ^26^ ^]^ The neurodegenerative conditions mainly involve the loss of photoreceptor cells. First, the progressive death of rods leads to nyctalopia in patients, which is followed by secondary degeneration of cones causing a total loss of light sensitivity.^[^ ^27^ ^]^ Knocking down the expression of orphan nuclear receptor NR2E3, which is necessary for rod development and maintenance, has been illustrated as a mutation‐independent treatment for RP, as loss of function in *Nr2e3* reprograms rods to a cone cell fate, therefore avoiding death caused by the rod‐specific disease‐causing mutations.^[^ ^28^, ^29^, ^30^ ^]^ Herein, we employed the widely‐used *Rho* ^P23H/+^ mice^[^ ^31^ ^]^ as the disease model of RP to conduct the following proof‐of‐concept research.

**Figure 3:**
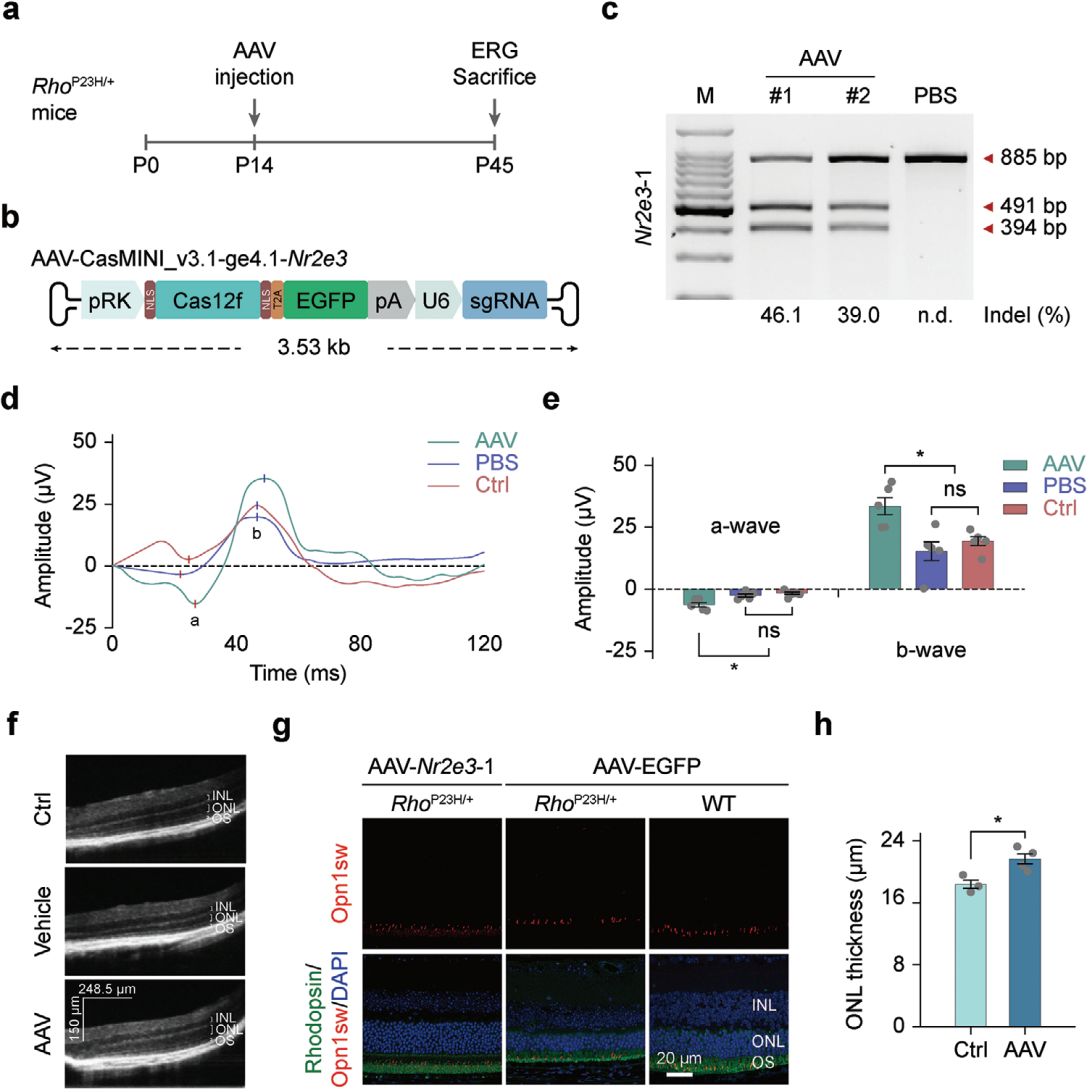
In vivo genome editing of *Nr2e3* gene rescues retinal degeneration in the *Rho^P23H/+^* mouse model of RP). a) Timeline for in vivo genome editing experiments in *Rho^P23H/+^* mice. Each mouse was subretinally injected with 1 × 10^9^ vector genomes (vg) of AAV8‐CasMINI_v3.1/ge4.1‐*Nr2e3* per eye. b) Schematic of the AAV construct encoding CasMINI_v3.1 and ge4.1 for in vivo gene editing. c) T7EI analysis of the indel frequencies generated by AAV8‐CasMINI_v3.1/ge4.1 on the *Nr2e3* target site 1 in mouse retinas. The indel rate is shown under the lanes with mutation. PBS, negative control with PBS injection. d) Representative ERG waveforms from *Rho^P23H/+^* mice treated with AAV8‐CasMINI_v3.1/ge4.1 (indicated as AAV), PBS, or uninjected (indicated as Ctrl). e) Quantification of light‐adapted a‐wave and b‐wave amplitude in AAV8‐CasMINI_v3.1/ge4.1‐*Nr2e3* injected (indicated as AAV), PBS injected (indicated as PBS), and uninjected (indicated as Ctrl) *Rho^P23H/+^* mice. Error bars indicate standard errors of the mean (s.e.m.), *n* = 5 (^*^ *p* < 0.05. Paired student's *t*‐test). f) Representative OCT scans showing increase in outer nuclear layer (ONL) thickness of the retina in *Rho^P23H/+^* mouse treated with AAV8‐CasMINI_v3.1/ge4.1‐*Nr2e3* (indicated as AAV‐*Nr2e3*‐1) in comparison with the PBS‐injected (indicated as Vehicle) *Rho^P23H/+^* mouse retina and untreated (indicated as Ctrl) *Rho^P23H/+^* mouse retina. g) Immunofluorescence staining of OPN1SW and RHODOPSIN on retinal sections from *Rho^P23H/+^* mice demonstrates preservation of retinal photoreceptor degeneration using *Nr2e3* knockout strategy via CasMINI_v3.1/ge4.1. h) Quantification of ONL thickness shows increased ONL thickness in AAV8‐CasMINI_v3.1/ge4.1‐*Nr2e3* injected *Rho^P23H/+^* mice, indicating rescued retinal degeneration using AAV‐Cas12f1‐based *Nr2e3* knockout strategy. Error bars indicate the s.e.m., *n* = 3 (^*^ *p* < 0.05. Paired student's *t*‐test).

*Rho* ^P23H/+^ mice were subretinally injected with AAVs carrying CasMINI_v3.1/ge4.1 to target *Nr2e3* site 1 (AAV8‐CasMINI_v3.1/ge4.1‐*Nr2e3*) at P14, before the onset of rod degeneration, and sacrificed at P45 post‐injection to evaluate the treatment performance (**Figure** **3**a). Notably, we used an updated version of AAV construct here in which the expression of Cas12f1 cassette is driven by the photoreceptor‐specific human rhodopsin kinase promoter (pRK)^[^ ^32^ ^]^ rather than the above‐used EF1α promoter (Figure 3b). Two independent retina samples were dissected and digested to harvest successfully transduced photoreceptor cells via FACS, which were further analyzed for gene mutagenesis frequencies. The T7EI assay and Sanger sequencing results demonstrated high on‐target editing efficiencies in *Rho* ^P23H/+^ mice (Figure 3c; Figure S4a,b, Supporting Information), consistent with results from wild‐type mice. We next investigated whether therapeutic benefits could be achieved in the RP model mice after treatment. At 1 month post injection, we first performed electroretinography (ERG) to assess the electrical activity of cone photoreceptors (photopic response). Encouragingly, treatment with AAV8‐CasMINI_v3.1/ge4.1‐*Nr2e3* resulted in an improved cone function in *Rho* ^P23H/+^ mice, demonstrated by the significantly increased b‐wave values relative to both control (PBS injected and untreated) groups (Figure 3d,e). Meanwhile, optical coherence tomography (OCT) scan results demonstrated an increased outer nuclear layer (ONL) in the treated retina compared with PBS‐injected and uninjected *Rho* ^P23H/+^ retinas (Figure 3f). In line with the OCT results, histologic analyses by H&E staining and immunofluorescent staining showed that the ONL of the treated retina became significantly thicker than the untreated retina (Figure 3g,h; Figure S4c, Supporting Information), further indicating the rescue of retinal degeneration following *Nr2e3* knockout. According to previous reports, the underlying rationale for this presented therapy is that NR2E3 dysfunction would lead to the transition of rod cells into cone‐like cells, rendering them resistant to rod‐specific gene mutations and consequently preventing secondary cone loss.^[^ ^29^, ^30^ ^]^ Rod and cone photoreceptors use distinct photopigments to detect light: rod cells utilize rhodopsin (RHO) and cone cells utilize S‐opsin (OPN1SW), M‐opsin or L‐opsin, making them widely used markers to characterize these two types of photoreceptors. Notably, in our immunofluorescence staining results, we observed photoreceptor cells co‐expressing rod and cone biomarkers (RHO and OPN1SW) in both *Rho* ^P23H/+^ and wildtype mice after AAV8‐CasMIMIN‐v3.1‐ge4.1‐*Nr2e3* treatment, indicating the generation of cell fate reprogramming of photoreceptor cells (Figure 3g; Figure S4d, Supporting Information), which further supported the previous findings.^[^ ^29^ ^]^ Collectively, these data revealed the therapeutic benefits achieved by targeted gene editing with AAV8‐CasMINI_v3.1/ge4.1‐*Nr2e3*.

### 2.4 Minimal Off‐Target Effects of CasMINI_v3.1/ge4.1 Editing

**Figure 4:**
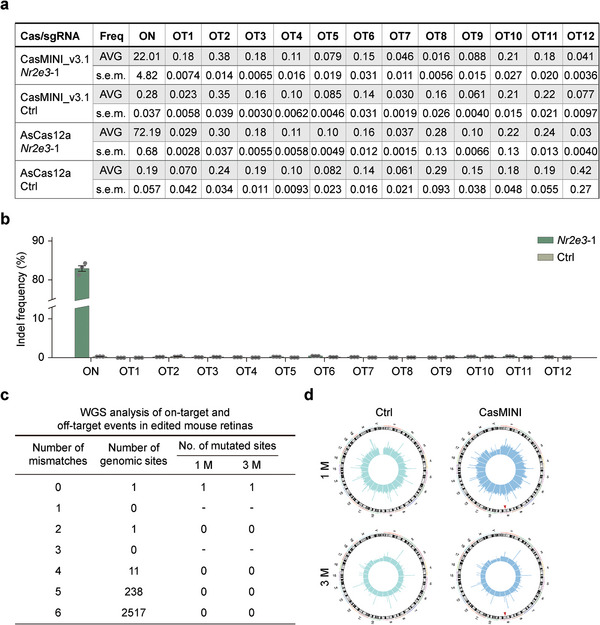
Minimal off‐target effects of CasMINI_v3.1/ge4.1‐mediated genome editing in vitro and in vivo. a) Off‐target frequency analyses of AsCas12a and CasMINI_v3.1/ge4.1 at in silico predicted off‐target sites of the mouse *Nr2e3* target site 1 in mouse N2A cells, determined by targeted deep sequencing. s.e.m., standard errors of the mean. *n* = 3. b) Off‐target frequency analyses of CasMINI_v3.1/ge4.1 at in silico predicted off‐target sites of the mouse *Nr2e3* target site 1 in mouse retinas, determined by targeted deep sequencing. Error bars indicate the standard error of the mean (s.e.m.), *n* = 3. c) WGS analysis for potential off‐target sites with one to nine mismatches to the mouse *Nr2e3* target site 1 in mouse retinal cells at 1 month (1 m) and 3 m after transduced with AAV8‐CasMINI_v3.1/ge4.1‐*Nr2e3*. d) WGS analysis for the translocation distribution patterns across the whole genome after AAV‐CasMINI_v3.1/ge4.1 infection at the *Nr2e3* site 1 in mouse retinas at 1 and 3 m. Red triangles indicate the *Nr2e3* target site.

The potential for off‐target gene editing has been a significant concern for CRISPR‐Cas systems with regard to their applications in gene therapy. We next investigated the gene editing specificity of the CasMINI_v3.1/ge4.1 system both in vitro and in vivo. We first assessed the in vitro specificity of CasMINI_v3.1/ge4.1 system in comparison with that of the CRIPSR‐AsCas12a system. Since both Cas12f1 and Cas12a nucleases recognize the 5′‐TTTN PAMs,^[^ ^16^, ^33^ ^]^ we designed two crRNAs for AsCas12a to target the same sites on the *Nr2e3* locus as CasMINI_v3.1 (Table S1, Supporting Information). Genomic loci with the potential for off‐target editing were predicted using Cas‐OFFinder.^[^ ^34^ ^]^ We selected potential off‐target sites with up to 4 nucleotide mismatches (Table S2, Supporting Information), and validated for the detection of off‐target indels with the use of targeted deep sequencing in edited N2A cells. Among all potential off‐target (OT) sites, minimal off‐target activities were detected (**Figure** **4**a; Figure S5a, Supporting Information), suggesting that CasMINI_v3.1/ge4.1 system is highly specific. Meanwhile, consistent with previous reports,^[^ ^22^ ^]^ our results also indicated that CasMINI_v3.1/ge4.1 shows relatively lower editing efficiency at the two target sites compared to AsCas12a/crRNA (Figure 4a; Figure S5a, Supporting Information). These results were in line with data from previous studies showing that CRISPR‐Cas12f1 nucleases, similar to Cas12a nucleases, exhibited minimal off‐target effects in vitro.^[^ ^11^, ^13^, ^22^ ^]^ However, in vivo assessment of the specificity of Cas12f1 nucleases is lacking.^[^ ^35^ ^]^ To address this issue, we next evaluated the potential in vivo off‐target effects by conducting targeted deep sequencing and WGS using isolated genomic DNA from infected retinal cells in mice at 1 month post subretinal injection. Both targeted deep sequencing and WGS results showed that the *Nr2e3* target site 1 is efficiently edited in AAV8‐CasMINI_v3.1/ge4.1‐*Nr2e3*‐injected (AAV‐injected) group (Figure 4b; Figure S5b, Supporting Information). Importantly, none of the 12 in silico predicted off‐target sites showed evidence of off‐target editing in AAV‐injected mouse retinas using targeted deep sequencing analysis (Figure 4b). Closely following that, the WGS data drew a consistent conclusion (Figure 4c). Meanwhile, WGS was carried out to detect structural variations in the *Nr2e3*‐edited mouse retinas, and no detectable chromosomal rearrangements such as large deletion and translocations were observed (Figure 4d; Figure S5c, Supporting Information). This further confirmed the ability of this Cas nuclease system to preserve genome integrity during gene editing. Together, our data demonstrates that the CasMINI_v3.1/ge4.1 system enables highly specific genome editing both in vitro and in vivo.

## 3 Discussion

In just a decade, the CRISPR gene editing has gone from the first demonstration of efficacy in human cells to several clinical trials,^[^ ^36^, ^37^ ^]^ showing unprecedented potential to treat a wide variety of genetic diseases and cancer. Nevertheless, only a few therapeutic options are available now as most genetic diseases require in vivo editing, and the efficient delivery of CRISPR‐Cas systems into mammalian tissues continues to play a critical role in advancing this field. The viral delivery of Cas effectors has been complicated by their large size and the limited packaging capacity of AAVs. To overcome this bottleneck, dual‐AAV approaches based on *trans*‐splicing inteins and the split Cas systems were adopted to address this problem. However, the split Cas system is usually less active, and the co‐delivery of two AAV vectors could reduce the delivery efficiency.^[^ ^1^, ^38^ ^]^ The recently identified Cas12f1 family nucleases are exceptionally hypercompact in size (with the engineered CasMINI_v3.1 effector used in our research being only 529 amino acids) and are packageable in a single AAV particle,^[^ ^12^, ^13^, ^14^, ^17^, ^20^ ^]^ which therefore endows this Cas system huge natural advantages to be applied in in vivo genome engineering.

In this study, we synthesized a set of Cas12f1 family proteins and sgRNA scaffolds engineered in previous studies,^[^ ^11^, ^12^, ^17^ ^]^ and systematically evaluated the pairwise targeting efficiencies of different combinations of Cas12f1 nucleases with sgRNA scaffolds. We found that CasMINI_v3.1/ge4.1 achieved the most consistent editing activity in both in vitro and in vivo assessments. When being packaged into single AAV vectors and injected subretinally, this CasMINI_v3.1/ge4.1 system yielded robust and over 70% editing efficiencies in infected retinal cells. Moreover, as a proof‐of‐concept for retinal gene therapy, we used this AAV‐Cas12f1 system to treat RP, a most prevalent inherited retinal disease in the most commonly used *Rho* ^P23H/+^ mouse model. Excitingly, by targeting *Nr2e3* gene via all‐in‐one AAV delivery of the CasMINI_v3.1/ge4.1 system, we successfully achieved therapeutically relevant levels of editing in the retina, and observed partially rescued outer layer structure and visual function. Moreover, we provided the first characterization of the in vivo specificity analysis of the engineered Cas12f1 nuclease and demonstrated that CasMINI_v3.1/ge4.1 exhibits minimal undesired bystander editing upon a persistent in vivo activity of the Cas12f1 nuclease.

As CRISPR‐based therapies are making remarkable strides with additional in vivo gene editing therapies moving toward the clinic, the availability of robust in vivo gene editing tools will be critical. The results from our study demonstrate that the CasMINI_v3.1/ge4.1 platform with high fidelity and efficiency is amenable to in vivo genome editing and is attractive to be leveraged for therapeutic benefits using the highly desirable all‐in‐one AAV delivery platform. Notably, the high‐performance CasMINI_v3.1/ge4.1 editing platform screened out in this study were both engineered variants from the originally ineffective CRISPR‐Un1Cas12f1 system.^[^ ^11^, ^17^ ^]^ Rational engineering of the effector protein and guide RNA optimization were also proved remarkably effective in enhancing the editing activities of different Cas12f1 systems, including OsCas12f1, RhCas12f1, and AsCas12f1, by enhancing the interaction of Cas effectors with sgRNA and increasing sgRNA stability.^[^ ^13^, ^20^, ^21^, ^39^, ^40^ ^]^ Moreover, the RhCas12f1 and CnCas12f1 discovered very recently could use 5′C‐rich PAMs,^[^ ^13^, ^41^ ^]^ which further expands the target range for editing the mammalian genome and broadens the range of treatable diseases by the miniature CRISPR‐Cas12f1 systems.

Importantly, in our study, we provided evidence showing that a single subretinal injection of the AAV‐delivered CasMINI_v3.1/ge4.1 system in a mouse model of RP could efficiently knockout *Nr2e3*, achieve therapeutic levels of gene editing, and lead to ERG improvement and structural preservation. Along with a recent in vivo study employing new Cas12f1 orthologs,^[^ ^13^ ^]^ this proof‐of‐concept study established the efficacy of Cas12f1‐based treatments in vivo in disease mouse models. Moreover, a significant concern in therapeutic CRISPR‐based gene editing is the possibility of off‐target edits. Early data has indicated that the high targeting specificity of Cas12f1 system ensures the safety of Cas12f1‐based editing activities in mammalian cells.^[^ ^11^, ^13^, ^22^ ^]^ In this study, we assessed the specificity of the CasMINI_v3.1/ge4.1 platform using both in vitro and in vivo edited retinal samples by targeted deep sequencing and WGS analyses, and a minimal off‐target profile was revealed, which further validated the safety property of this system. As the breadth of diseases addressable by genome editing is widening, the development of Cas12f1‐based therapeutic approaches for a range of human conditions might also be greatly accelerated.

Overall, our results highlighted the miniature Cas12f1 system as a promising gene editing tool for therapeutic applications that combines key advantages of hypercompact size, high in vivo editing efficiency, and minimal off‐target effects.

## 4 Experimental Section

### Plasmid Construction

Human codon‐optimized genes encoding Un1Cas12f1 and AsCas12f1 (Supplementary Sequences, Supporting Information) were *de novo* synthesized and constructed into a pCAG‐2AeGFP backbone vector as the previous report.^[^ ^42^ ^]^ Un1Cas12f1 mutant, CasMINI_v3.1, was generated by site‐directed mutagenesis (Table S3 and Supplementary Sequences, Supporting Information). Guide RNA (gRNA) scaffolds (ge4.1, ge4.0, Design2, AsgRNA) (Supplementary Sequences, Supporting Information) were *de novo* synthesized and constructed into a pUC19‐U6 backbone vector.^[^ ^42^ ^]^ Targeting gRNAs for cell transfection was constructed by ligating annealed oligos into BsaI‐digested pUC19‐U6‐gRNA vectors.

### Adeno‐Associated Virus (AAV) Construction and Production

CasMINI_v3.1, 2AEGFP‐pA, U6‐ge4.1‐*Nr2e3* fragments were amplified using overlapping primers (Table S3, Supporting Information), and constructed into AvrII + XbaI digested pAAV‐EF1α backbone (Supplementary Sequences, Supporting Information) using NEBuilder HiFi DNA Assembly (NEB) according to the manufacturer's instruction. To change EF1α promoter into pRK promoter, the pRK fragment was amplified using overlapping primers (Table S3, Supporting Information), and constructed into XhoI + AvrII digested pAAV‐CasMINI_v3.1‐ge4.1‐*Nr2e3* backbone via DNA assembly cloning. AAVs were produced by PackGene Biotech (Guangzhou, China).

### Cell Lines and Transfection

The Neuro‐2a (N2A) cells were cultured in Dulbecco's Modified Eagle Medium (DMEM, Gibco) supplemented with 10% (v/v) fetal bovine serum (FBS, Gibco), 1% penicillin and streptomycin (Gibco) at 37 °C with 5% CO_2_ incubator. According to the manufacturer's instructions of jetPRIME (Polyplus Transfection, 114–15), 2 × 10^5^ Neuro‐2a cells were plated onto a 24‐well plate and transfected with plasmids encoding a CRISPR‐Cas nuclease (500 ng) and the cognate sgRNA (250 ng). Then 72 h following transfection, GFP‐positive cells were sorted using the MoFlo XDP (Beckman Coulter) for further analysis.

### T7 Endonuclease I (T7EI) Assay and Sanger Sequencing

Genomic DNA was isolated from GFP‐positive N2A cells using the Mouse Direct PCR Kit (Selleck). Target‐specific primers (Table S4, Supporting Information) were used to amplify protospacer‐containing regions with Q5 Hot Start High‐Fidelity 2X Master Mix (NEB) according to the manufacturer's instructions with modifications. The PCR amplicons were re‐annealed for T7EI cleavage assay, and T7EI‐identified mutated products were cloned to pEASY‐Blunt cloning vector (Transgen) for Sanger sequencing.^[^ ^42^, ^43^ ^]^

### Targeted Deep Sequencing

Potential off‐target sites with less than four mismatches for CRISPR‐Cas systems were predicted using Cas‐OFFinder.^[^ ^34^ ^]^ The short PCR products were amplified using designed primers (Tables S5 and S6, Supporting Information), and pooled libraries were subjected to paired‐end sequencing using NovaSeq (Illumina). Cutadapt and Seqtk (https://github.com/lh3/seqtk) were applied to separate the fastq file into different groups. NovaSeq paired‐end reads were mapped to mouse genome reference using BWA and mutation analyses were conducted using CRISPResso2.^[^ ^44^ ^]^

### Animals

The C57BL/6 mice and *Rho* ^P23H/+^ mice were housed in the animal care facility of the Institute of Zoology, Chinese Academy of Sciences. All animal experiments were performed according to the guidelines for the care and use of laboratory animals established by the Beijing Association for Laboratory Animal Science and approved under the Animal Ethics Committee of the Institute of Zoology, Chinese Academy of Sciences (approval number: IOZ‐IACUC‐2021‐195)

### Subretinal Injection

Mice were anesthetized by intraperitoneal injection of Tribromoethanol (150 mg kg^−1^). Pupils were subsequently dilated with tropicamide (0.5%) and phenylephrine hydrochloride (0.5%). A small hole was made through the cornea limbus at the Temporal side using a 30‐gauge needle (BD Biosciences, 305106) under a stereoscope. A 19 mm 33‐gauge blunt‐end needle (Hamilton 7803–05) fitted to a Hamilton syringe (Hamilton, 7633‐01) was then inserted through the tunnel. One microliter of AAV particles or PBS was then injected slowly into the subretinal space. Fluorescein (100 mg mL^−1^, Alcon Laboratories, lnc) was included in the suspensions (0.1% by volume). Carbomer Eye Gel (Carbomer, 0.2%, Alcon Laboratories, lnc) was applied to each eye after the surgery. Mice showing any sign of retinal damage such as bleeding were discarded and excluded from the subsequent study.

### Immunofluorescence

Mouse eyes were collected immediately after the killing. Detached retinas were fixed in ocular fixative (Servicebio G1109) for 24 h, dehydrated (75%, 85%, 90%, 95%, 100% ethanol, alcohol benzene, and xylene), and embedded in paraffin. Three micrometers of retinal sections were prepared using a Leica slicing machine (Leica Biosystems, RM2016). Immunofluorescence staining was performed after dewaxing and hydration. The retinal sections were blocked using blocking buffer (1x PBS, 2.5% BSA, and 5% donkey serum) for 45 min, and then incubated with primary antibodies diluted in blocking buffer at 4 °C overnight. After washing three times in PBS, sections were incubated with secondary antibodies and Hoechst 33342 at 37 °C for 1 h. Images were captured by an LSM880 confocal laser scanning microscope (Zeiss, Germany). Primary and secondary antibodies used in this study are listed in Table S7 (Supporting Information).

### Hematoxylin‐Eosin Staining and ONL Thickness Analysis

For HE‐staining, the eyeball sections were rehydrated in xylene and alcohol. To observe cell nuclei, sections were stained with hematoxylin for 3–5 min and rinsed with running water for 5 min. After adding the acid ethanol for 10 s, the samples were rinsed with running water for 5 min again. The staining of the nuclei could be observed under a microscope at this point. To visualize the cytoplasm, the samples were stained with eosin (alcohol‐soluble) for 15–30 s and sequentially dehydrated in a gradient of alcohol and xylene. The Canada Balsam‐sealed (Solarbio, #C8300) samples were scanned using a panoramic histocytometer (Leica Aperio VESA8, Germany). For each eye, the ONL thickness at three random sites was measured and averaged using ImageScope.

### Optical Coherence Tomography (OCT)

An Ultramicro Ophthalmol Imaging System (ISOCT, Optoprobe Science LTD) was used to image the retina before and after injection at indicated time intervals. Anesthetized mice were placed on the instrument with pupils dilated. The retinas were scanned with OCT after the light source was focused.

### Electroretinogram

The visual function of model mice was analyzed by Visual Electrophysiology Instrument (ERG, Optoprobe Science LTD). Mice were anesthetized by intraperitoneal injection of Tribromoethanol (150 mg kg^−1^) and pupils were subsequently dilated with tropicamide (0.5%) and phenylephrine hydrochloride (0.5%). Small silver wire loops were placed on each cornea. Both cheeks were injected with silver needles reference electrodes, and a ground electrode was placed subcutaneously in the tail. The cone cell response (white light: 600 cd.m^−2^, flash stimulation: 5 ms, Flash intensity: 3.0 cd.s.m^−2^, stimulation interval: 1s) was recorded.

### Retinal Dissociation and Flow Cytometry

Mouse eyeballs were dissected out after sacrifice. Then retinas were detached and dissociated using the Papain Dissociation System (Worthington, LK003150). The cell suspension was sorted and analyzed by flow cytometry (BD fusion), with propidium iodide (PI) staining to exclude dead cells.

### Whole Genome Sequencing (WGS)

Qualified genomics DNA from GFP‐positive retinal cells was sequenced using an Illumina NovaSeq sequencer at a sequencing depth of 30x diploid coverage. BWA (v0.7.17) was used to map qualified sequencing reads to the mouse reference genome (mm10) and SamTools (v1.9) was applied to sort the mapped BAM files. Duplicates in the sorted BAM file were removed using Picard Tools MarkDuplicates.^[^ ^45^ ^]^ GATK (v4.1.4.0) was used to apply Base Quality Score Recalibration. Mutect2 was applied to call somatic mutation with tumor‐normal mode and FilterMutectCalls was applied to filter the mutation.^[^ ^46^ ^]^ SNVs near low‐complexity regions that were defined by RepeatMasker (http://repeatmasker.org) were removed and annotated by soft masking in 10 mm. Then SNVs and indels that cause expansions or compressions of long (≥ 5 bp) homopolymers were removed. For off‐target analysis, genomic loci with potential for off‐target editing were found using Cas‐OFFinder,^[^ ^34^ ^]^ and further validated for the detection of overlapping indels and SNVs. The chromosome sequencing depth distribution chart displays the distribution of sequencing depths along the chromosomes, with a maximum depth limit of 100. The chromosomal sequences were divided into 500 kb bins, and the chart was generated using RCircos. Raw data has been deposited in the Genome Sequence Archive (GSA) in the National Genomics Data Center, China National Center for Bioinformation/Beijing Institute of Genomics, Chinese Academy of Sciences (GSA: CRA013195) that are publicly accessible at https://ngdc.cncb.ac.cn/gsa.

### Statistical Analysis

Statistical analyses were performed using GraphPad Prism software. All data are presented as mean ± S.E.M. Each experiment included at least three independent samples. Comparison between the two groups was made by Student's two‐tailed *t*‐test. All statistical analyses of experimental *n* numbers and *p* values are described in the figure legends. *p* < 0.05 was considered to indicate a significant result.

## Conflict of Interest

The authors declare no conflict of interest.

## Author Contributions

T.C. and B.C. contributed equally to this work. F.T., W.L., Q.Z., and T.C. conceived the project and designed the experiments; T.C., F.T., B.C., X.L., C.L., Q.G., B.L., and R.L. performed the experiments; F.T., T.C., B.C., Y.T., and Y.D. analyzed the data; F.T., W.L., and T.C. wrote the manuscript with assistance from other authors.

## Supporting information

Supporting Information

## Data Availability

The data that support the findings of this study are available from the corresponding author upon reasonable request.
