# Supplementary material for: Therapeutic In Vivo Gene Editing Achieved by a Hypercompact CRISPR‐Cas12f1 System Delivered with All‐in‐One Adeno‐Associated Virus
Source: Adv Sci (Weinh). 2024 Feb 26;11(19):2308095. doi: 10.1002/advs.202308095 (PMC11109646; doi:10.1002/advs.202308095)
Supplement: Supplementary file 1 — Supporting Information [file ADVS-11-2308095-s001.pdf]

ADVANCED SCIENCE
Open Access

Supporting Information

for *Adv. Sci.*, DOI 10.1002/advs.202308095

Therapeutic In Vivo Gene Editing Achieved by a Hypercompact CRISPR-Cas12f1 System Delivered with All-in-One Adeno-Associated Virus

Tongtong Cui, Bingyu Cai, Yao Tian, Xin Liu, Chen Liang, Qingqin Gao, Bojin Li, Yali Ding, Rongqi Li, Qi Zhou, Wei Li and Fei Teng**

Supporting Information

This file includes:

Supplementary Figure 1
Supplementary Figure 2
Supplementary Figure 3
Supplementary Figure 4
Supplementary Figure 5
Supplementary Table 1
Supplementary Table 2
Supplementary Table 3
Supplementary Table 4
Supplementary Table 5
Supplementary Table 6
Supplementary Table 7
Supplementary Sequences

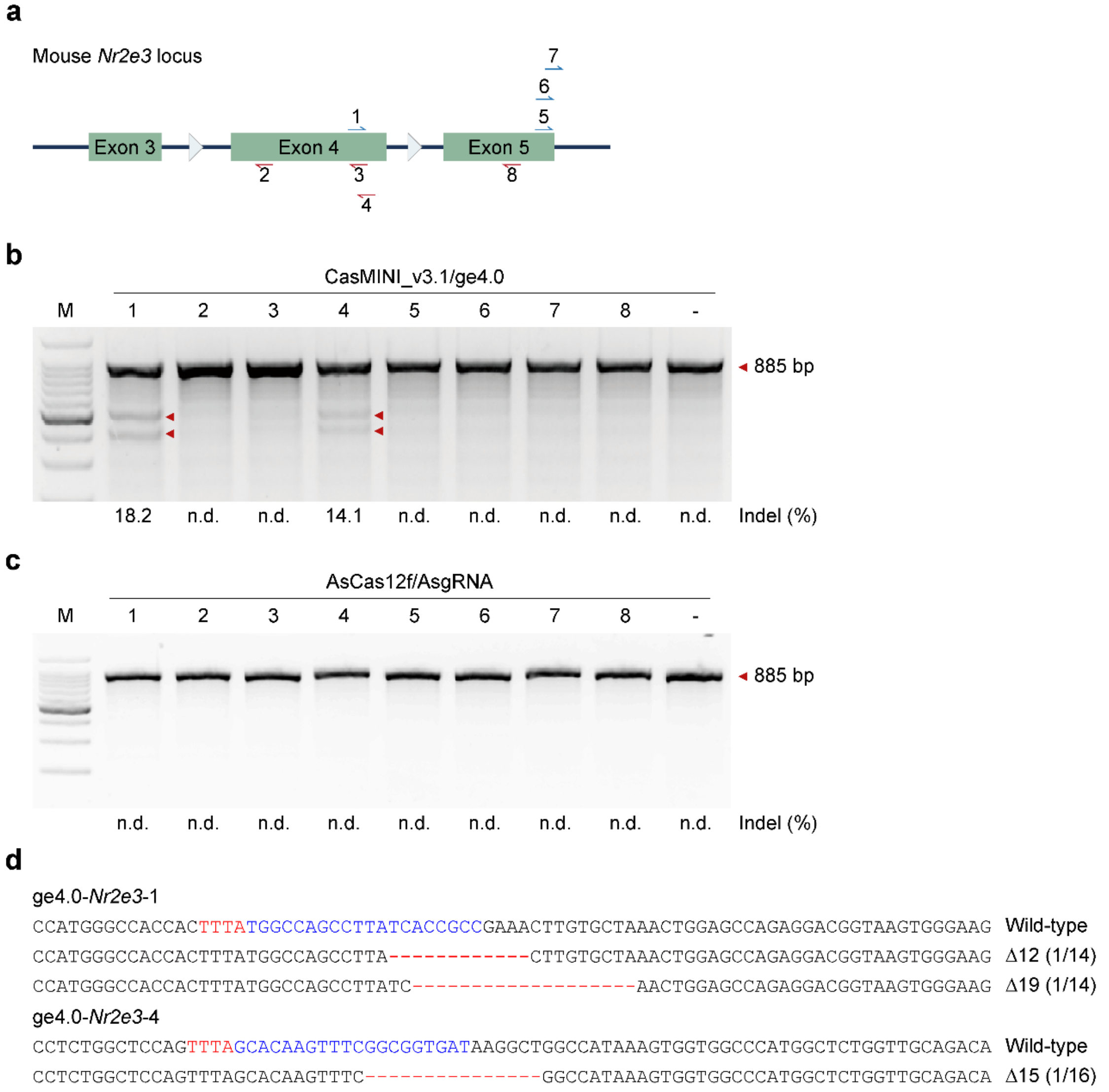

Figure S1. Screening of optimized sgRNAs for mouse *Nr2e3* gene editing. a) Schematic illustration of eight target sites of Cas12f1 systems in the mouse *Nr2e3* locus. b, c) T7EI analysis of eight sgRNAs targeting the mouse *Nr2e3* gene using the CasMINI_v3.1/ge4.0 (b) and AsCas12f1/AsgRNA (c) systems. The indel rate is shown under the lanes with mutation. -, U6 empty vector without gRNA expression. d) Sanger sequencing results illustrating representative indels on the mouse *Nr2e3* target site 1 and 4 produced by CasMINI_v3.1/Design2. Red dashes, deleted bases; red uppercases, PAM; blue uppercases, protospacer.

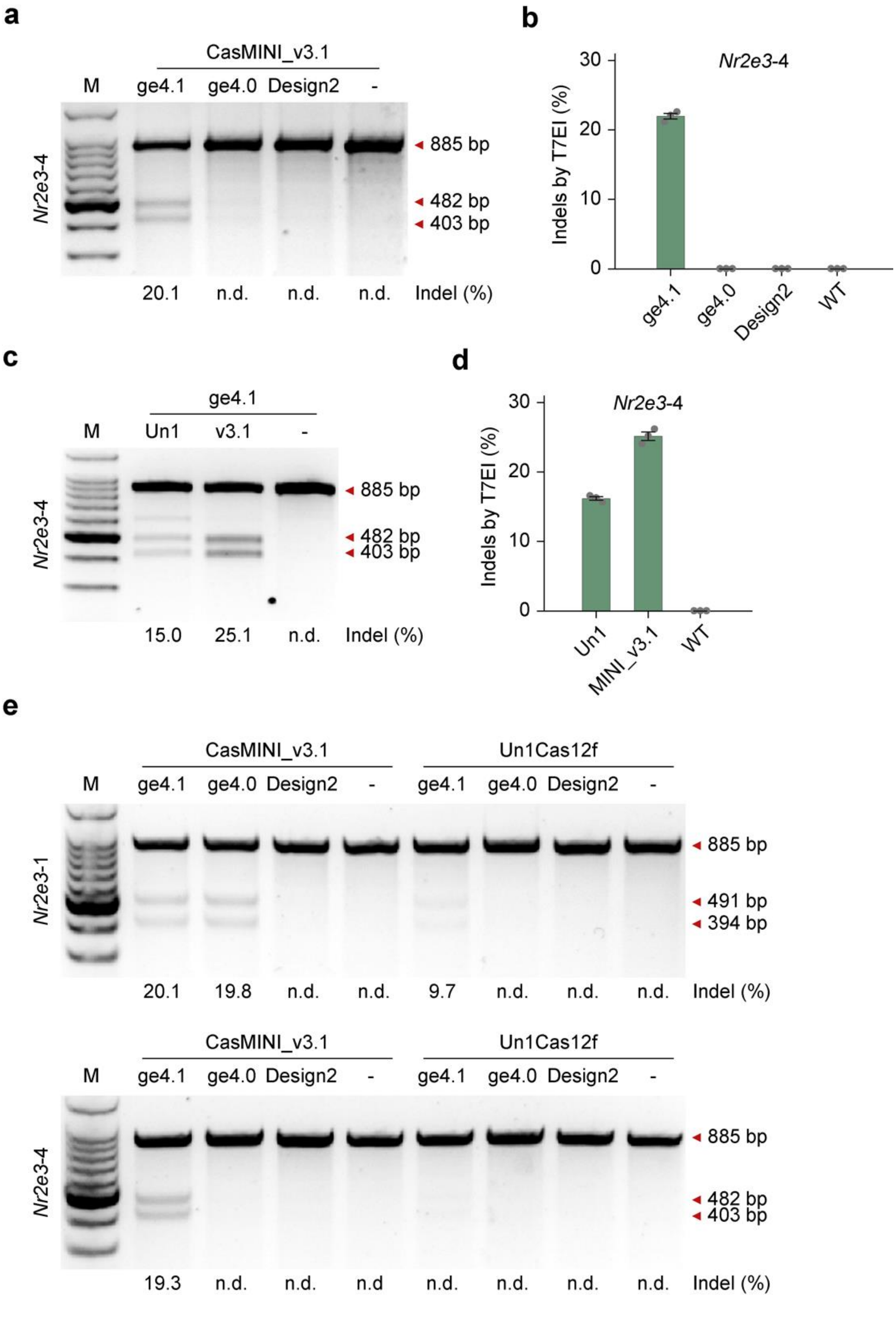

Figure S2. Optimizing pairwise Cas12f1/sgRNA combinations for genome editing. a) T7EI analysis of the indels generated by CasMINI_v3.1 with different sgRNA scaffolds (ge4.1, ge4.0, Design2) on the mouse *Nr2e3* target site 4. The indel rate is shown under the lanes with mutation. -, U6 empty vector without sgRNA expression. b) Indel frequencies of different sgRNA scaffolds on the mouse *Nr2e3* target site 4 in mouse N2A cells, determined by T7EI assay. Error bars indicate the standard error of the mean (s.e.m.), n = 3. c) T7EI analysis of the indels generated by Un1Cas12f1 and CasMINI_v3.1 with sgRNA scaffolds ge4.1 on the mouse *Nr2e3* target site 4. The indel rate is shown under the lanes with mutation. -, pCAG-2AeGFP empty vector without Cas12f1 expression. d) Indel efficiency comparison between Un1Cas12f1 and CasMIN_v3.1 on the mouse *Nr2e3* target site 4 in mouse N2A cells, determined by T7EI assay. Error bars indicate the standard error of the mean (s.e.m.), n = 3. e) Indel frequencies of Cas12f1

(Un1Cas12f1 and CasMIN_v3.1) with different sgRNA scaffolds (ge4.1, ge4.0, Design2) on the mouse *Nr2e3* target sites 1 and 4 in mouse N2A cells, determined by T7EI assay.

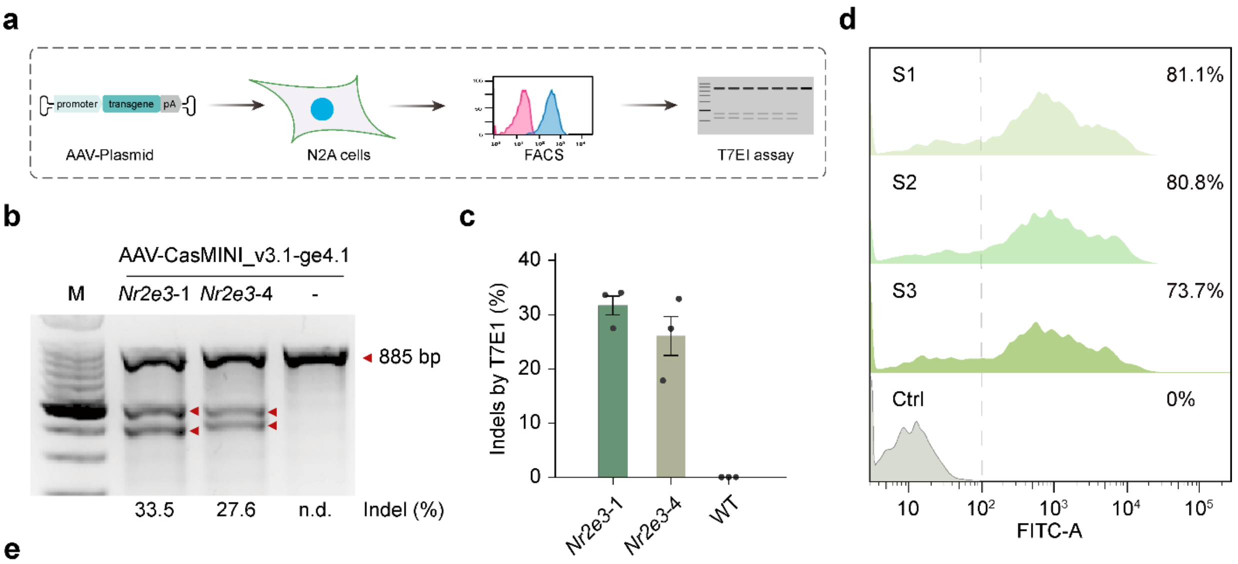

e

Mouse #1

```
CCATGGGCCACCACTTTATGGCCAGCCTTATCACCGCCGAAACTTGTGCTAAACTGGAGCCAGAGGACGGTAAGTGGGAAG  Wild-type
CCATGGGCCACCACTTTATGGCCAGCCTTATCAC---------TTGTGCTAAACTGGAGCCAGAGGACGGTAAGTGGGAAG  Δ9 (5/21)
CCATGGGCCACCACTTTATGGCCAGCCTTATCAC----------TGTGCTAAACTGGAGCCAGAGGACGGTAAGTGGGAAG  Δ10 (1/21)
CCATGGGCCACCACTTTATGGCCAGCCTTATCA----------TTGTGCTAAACTGGAGCCAGAGGACGGTAAGTGGGAAG  Δ10 (2/21)
CCATGGGCCACCACTTTATGGCCAGCCTTA-----------ACTTGTGCTAAACTGGAGCCAGAGGACGGTAAGTGGGAAG  Δ11 (1/21)
CCATGGGCCACCACTTTATGGCCAGCCTTATCAC-----------GTGCTAAACTGGAGCCAGAGGACGGTAAGTGGGAAG  Δ11 (1/21)
CCATGGGCCACCACTTTATGGCCAGCCTTA------------CTTGTGCTAAACTGGAGCCAGAGGACGGTAAGTGGGAAG  Δ12 (1/21)
CCATGGGCCACCACTTTATGGCCAGCCTTA--------------TGTGCTAAACTGGAGCCAGAGGACGGTAAGTGGGAAG  Δ14 (1/21)
CCATGGGCCACCACTTTATGGCCAGCCTT--------------TTGTGCTAAACTGGAGCCAGAGGACGGTAAGTGGGAAG  Δ14 (2/21)
CCATGGGCCACCACTTTATGGCCAGCCTTATCAC--------------CTAAACTGGAGCCAGAGGACGGTAAGTGGGAAG  Δ14 (1/21)
CCATGGGCCACCACTTTATGGCCAGCCTTATCAC---------------TAAACTGGAGCCAGAGGACGGTAAGTGGGAAG  Δ15 (1/21)
CCATGGGCCACCACTTTATGGCCAGCCTT----------------GTGCTAAACTGGAGCCAGAGGACGGTAAGTGGGAAG  Δ16 (1/21)
CCATGGGCCACCACTTTATGGCCAGCCTTA-------------------TAAACTGGAGCCAGAGGACGGTAAGTGGGAAG  Δ19 (1/21)
CCATGGGCCACCACTTTATGGCCAGCCTTA---------------------AACTGGAGCCAGAGGACGGTAAGTGGGAAG  Δ21 (1/21)
```

Mouse #2

```
CCATGGGCCACCACTTTATGGCCAGCCTTATCACCGCCGAAACTTGTGCTAAACTGGAGCCAGAGGACGGTAAGTGGGAAG  Wild-type
CCATGGGCCACCACTTTATGGCCAGCCTTATCAC---------TTGTGCTAAACTGGAGCCAGAGGACGGTAAGTGGGAAG  Δ9 (4/18)
CCATGGGCCACCACTTTATGGCCAGCCTTATCAC----------TGTGCTAAACTGGAGCCAGAGGACGGTAAGTGGGAAG  Δ10 (1/18)
CCATGGGCCACCACTTTATGGCCAGCCTTATCA-----------TGTGCTAAACTGGAGCCAGAGGACGGTAAGTGGGAAG  Δ11 (1/18)
CCATGGGCCACCACTTTATGGCCAGCCTTA------------CTTGTGCTAAACTGGAGCCAGAGGACGGTAAGTGGGAAG  Δ12 (1/18)
CCATGGGCCACCACTTTATGGCCAGCCTTAT-------------TGTGCTAAACTGGAGCCAGAGGACGGTAAGTGGGAAG  Δ13 (1/18)
CCATGGGCCACCACTTTATGGCCAGCCTTATC-------------GTGCTAAACTGGAGCCAGAGGACGGTAAGTGGGAAG  Δ13 (1/18)
CCATGGGCCACCACTTTATGGCCAGCCTTATCACC--------------TAAACTGGAGCCAGAGGACGGTAAGTGGGAAG  Δ14 (1/18)
CCATGGGCCACCACTTTATGGCCAGCCTTAT--------------GTGCTAAACTGGAGCCAGAGGACGGTAAGTGGGAAG  Δ14 (1/18)
CCATGGGCCACCACTTTATGGCCAGCCTTATCAC----------------AAACTGGAGCCAGAGGACGGTAAGTGGGAAG  Δ16 (1/18)
CCATGGGCCACCACTTTATGGCCAGCCTTATCA-----------------AAACTGGAGCCAGAGGACGGTAAGTGGGAAG  Δ17 (1/18)
CCATGGGCCACCACTTTATGGCCAGCCTTAT------------------------GGAGCCAGAGGACGGTAAGTGGGAAG  Δ24 (1/18)
CCATGGGCCACCACTTTATGG-------------------------TGCTAAACTGGAGCCAGAGGACGGTAAGTGGGAAG  Δ25 (1/18)
```

Mouse #3

```
CCATGGGCCACCACTTTATGGCCAGCCTTATCACCGCCGAAACTTGTGCTAAACTGGAGCCAGAGGACGGTAAGTGGGAAG  Wild-type
CCATGGGCCACCACTTTATGGCCAGCCTTATCAC---------TTGTGCTAAACTGGAGCCAGAGGACGGTAAGTGGGAAG  Δ9 (6/20)
CCATGGGCCACCACTTTATGGCCAGCCTTATCACC---------TGTGCTAAACTGGAGCCAGAGGACGGTAAGTGGGAAG  Δ9 (1/20)
CCATGGGCCACCACTTTATGGCCAGCCTTAT----------ACTTGTGCTAAACTGGAGCCAGAGGACGGTAAGTGGGAAG  Δ10 (1/20)
CCATGGGCCACCACTTTATGGCCAGCCTTATCAC-----------GTGCTAAACTGGAGCCAGAGGACGGTAAGTGGGAAG  Δ11 (1/20)
CCATGGGCCACCACTTTATGGCCAGCCTTATCA-----------TGTGCTAAACTGGAGCCAGAGGACGGTAAGTGGGAAG  Δ11 (1/20)
CCATGGGCCACCACTTTATGGCCAGCCTTATC-----------TTGTGCTAAACTGGAGCCAGAGGACGGTAAGTGGGAAG  Δ11 (1/20)
CCATGGGCCACCACTTTATGGCCAGCCTTA------------CTTGTGCTAAACTGGAGCCAGAGGACGGTAAGTGGGAAG  Δ12 (2/20)
CCATGGGCCACCACTTTATGGCCAGCCTTATCAC---------------TAAACTGGAGCCAGAGGACGGTAAGTGGGAAG  Δ15 (1/20)
CCATGGGCCACCACTTTATGGCCAGCCTTATCACC------------------CTGGAGCCAGAGGACGGTAAGTGGGAAG  Δ18 (1/20)
CCATGGGCCACCACTTTATGGCCAGCCTTATC-----------------------------------CGGTAAGTGGGAAG  Δ35 (1/20)
```

Figure S3. Gene editing in mouse N2A cells using AAV plasmids. a) Schematic of CasMINI_v3.1/ge4.1-mediated genome editing using AAV plasmids in mouse cells. b) T7EI analysis of the indels generated by CasMINI_v3.1/ge4.1 on the mouse *Nr2e3* target site 1 and 4. The indel frequency is shown under the lanes with mutation. -, U6 empty vector

without sgRNA expression. c) CasMINI_v3.1/ge4.1-mediated indel frequencies on the mouse *Nr2e3* target sites 1 and 4 in mouse N2A cells, determined by T7EI assay. Error bars indicate the standard error of the mean (s.e.m.), $n = 3$. d) Assessment of the AAV infection efficiency indicated by the percentage of GFP-positive population values in fluorescence-activated cell sorting (FACS) assay. Samples from 3 individual mouse retinas were tested. e) Representative indels generated by AAV8-CasMINI_v3.1/ge4.1-*Nr2e3*-1 in mouse retinas. Three mouse retinas are genotyped by Sanger sequencing after gene editing. Red dashes, deleted bases; red uppercases, PAM; blue uppercases, protospacer.

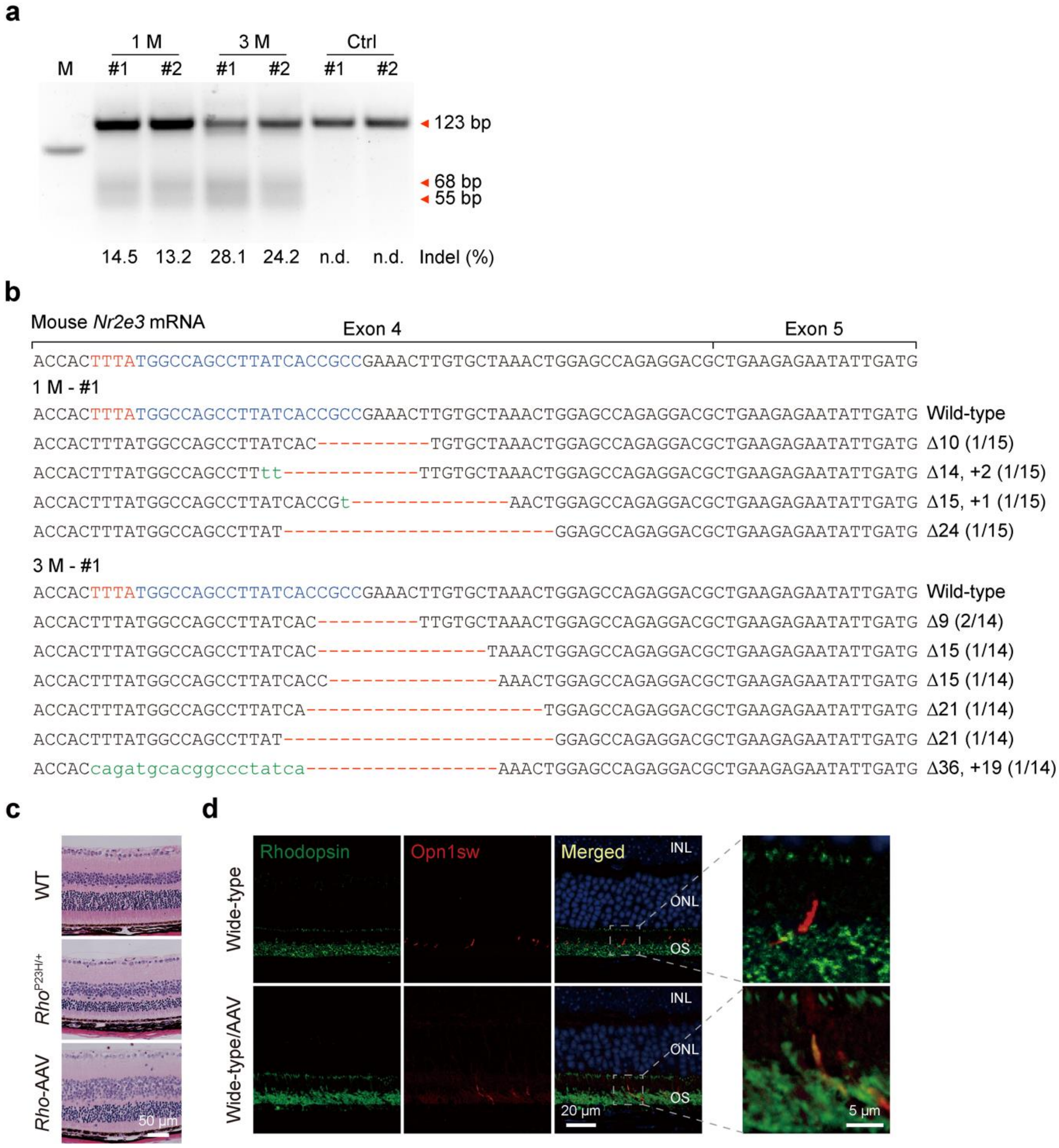

Figure S4. Efficient *in vivo* gene editing at the *Nr2e3* locus in mouse retinas. a) T7EI cleavage RT-PCR amplicons indicated induced mutations at the mRNA level in mouse retinas received AAV8-CasMINI_v3.1/ge4.1-*Nr2e3*-1 treatment at 1-month (1 M) and 3-month (3M) post injection. b) Sanger sequencing of RT-PCR products encompassing the *Nr2e3* site 1. c) H&E staining shows higher survival rate of photoreceptors with AAV8-CasMINI_v3.1/ge4.1-*Nr2e3*-1 treatment in *Rho*$^{P23H/+}$ mice. d) Co-expression of RHODOPSIN and OPN1SW indicates cell fate reprogramming in *Nr2e3* knockout cells in the wildtype mouse retinas.

a

Cas/gRNA	Freq	ON	OT1	OT2	OT3	OT4	OT5	OT6	OT7	OT8	OT9	OT10	OT11	OT12	OT13	OT14	OT15
CasMINI_v3.1 *Nr2e3*-4	AVG	11.26	0.055	0.19	0.16	0.13	0.048	0.087	0.096	0.071	0.27	0.055	0.050	0.16	0.21	0.21	0.15
	s.e.m.	4.60	0.0018	0.069	0.027	0.027	0.0023	0.016	0.0093	0.014	0.031	0.010	0.012	0.013	0.038	0.016	0.034
CasMINI_v3.1 Ctrl	AVG	0.33	0.089	0.13	0.23	0.12	0.097	0.95	0.087	0.096	0.36	0.092	0.073	0.15	0.23	0.34	0.098
	s.e.m.	0.0074	0.0058	0.0058	0.082	0.0097	0.0058	0.0026	0.0058	0.0032	0.018	0.0081	0.0030	0.0054	0.0037	0.068	0.0039
AsCas12a *Nr2e3*-4	AVG	74.24	0.079	0.16	0.30	0.11	0.060	0.11	0.094	0.11	0.26	0.13	0.092	0.18	0.17	0.33	0.11
	s.e.m.	1.32	0.0044	0.0029	0.13	0.013	0.0074	0.0039	0.0064	0.0021	0.0017	0.0024	0.0060	0.0093	0.018	0.075	0.0045
AsCas12a Ctrl	AVG	0.062	0.13	0.12	0.39	0.11	0.064	0.14	0.099	0.088	0.25	0.12	0.058	0.23	0.26	0.26	0.14
	s.e.m.	0.0070	0.087	0.0050	0.068	0.0032	0.0055	0.0075	0.0032	0.012	0.020	0.031	0.0060	0.030	0.040	0.038	0.011

b

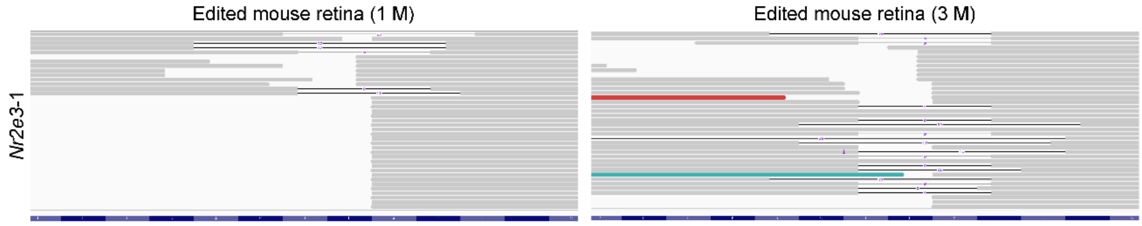

Figure S5. Off-target analysis in AAV8-CasMINI_v3.1/ge4.1-*Nr2e3*-1-treated mouse retinas. a) Analysis of potential off-target effects induced by AsCas12a and CasMINI_v3.1/ge4.1 *in vitro*. Off-target frequency of AsCas12a and CasMINI_v3.1/ge4.1 at *in silico* predicted off-target sites of the mouse the *Nr2e3* target site 4 in mouse N2A cells, determined by targeted deep sequencing. s.e.m., the standard error of the mean. *n* = 3. b) Confirmation of the on-target mutation at *Nr2e3* site 1 by analysis of WGS.

Table S1 Protospacer sequences of mouse *Nr2e3* target sites.

**CRISPR-Cas**	**Protospacer ID**	**Protospacer sequences (5'-3')**	**5'PAM**	**Strand**
Un1Cas12f1/ AsCas12f	*Nr2e3*-1	TGGCCAGCCTTATCACCGCC	TTTA	+
	Nr2e3-2	CATGGCATCCAGGTGGACCT	TTTC	-
	Nr2e3-3	GGCGGTGATAAGGCTGGCCA	TTTC	-
	Nr2e3-4	GCACAAGTTTCGGCGGTGAT	TTTA	-
	Nr2e3-5	CCAACCTGCCTTTCCGGGAC	TTTT	+
	Nr2e3-6	CAACCTGCCTTTCCGGGACC	TTTC	+
	Nr2e3-7	CGGGACCAGGTACACAGCAC	TTTC	+
	Nr2e3-8	ACAGCCATGAAGAGCAGGCG	TTTG	-
AsCas12a	*Nr2e3*-1	TGGCCAGCCTTATCACCGCC	TTTA	+
	Nr2e3-4	GCACAAGTTTCGGCGGTGAT	TTTA	-

Table S2 Potential off-target sites with one to four mismatches predicted by Cas-OFFinder.

Target ID	Off-target ID	Off-target sequence (5' – 3')	Chromosome	Mismatch	Strand
Nr2e3-1	OT1	TTTATGGCCAGCCTcATCACCGaC	chr15	2	+
	OT2	TTTGTGGCCtcCCTTgTCcCCGCC	chr5	4	+
	OT3	TTTTTGGCCAGCCTTgTCcCttCC	chr7	4	+
	OT4	TTTGaGGCCAGCCTagTCACaGCC	chr12	4	+
	OT5	TTTCTGGCtAGCCTTtTCtCCtCC	chr4	4	+
	OT6	TTTTTGGCtttCCTTATCACCaCC	chr16	4	-
	OT7	TTTCTGGCCAcagTaATCACCGCC	chr9	4	+
	OT8	TTTATGGaCAGgaTTATCAtCGCC	chr11	4	+
	OT9	TTTCTtGCCAGCCTcgTCACCaCC	chr11	4	-
	OT10	TTTGTGGCCtGCCTTATCcCaGCa	chr13	4	-
	OT11	TTTGaGGCCAGCCTTATCAaCaCa	chr19	4	+
	OT12	TTTATGaCCAGaCTTATCACCttC	chr3	4	+
Nr2e3-4	OT1	TTTGaCACAgGTTTgGGCtGTGAT	chr8	4	-
	OT2	TTTGcCACAAGTTcCGGgGGTGAT	chr5	3	+
	OT3	TTTGaCACAAGTTTCtGCcGTGAT	chr1	3	+
	OT4	TTTTGCACtAGgTTtGGtGGTGAT	chr1	4	+
	OT5	TTTAGCtCAAGTTTgGGCaGTaAT	chr7	4	+
	OT6	TTTAGCtCAtGTTTgGGCaGTGAT	chr2	4	+
	OT7	TTTGGCACAgGTcTCtGtGGTGAT	chr4	4	-
	OT8	TTTAGagCAAGTTTCGGCccTGAT	chr17	4	-
	OT9	TTTAGCAtAAtTTTgGGgGGTGAT	chr17	4	-
	OT10	TTTTaCACAAGTTTgGGaGGTGAa	chrX	4	+
	OT11	TTTAGCACAgGTTTCtGCtGTcAT	chrX	4	-
	OT12	TTTAGCtCAtGTTTgGGCaGTGAT	chrX	4	+
	OT13	TTTAGCACAAcTTTCGGCGcTGAc	chr19	3	+
	OT14	TTTAGCACAAcTTTCGGCGcTGAc	chr3	3	+
	OT15	TTTTGCACgAtTTTCcGCGGTGAT	chr3	3	-

Table S3 Overlapping primers used for AAV vector construction.

Primer ID	Primer sequences (5’ – 3’)	Length
CasMINI-1F	ATGCCCAAGAAGAAGCGCAAGGTGCCCGGGATGGCCAAAAACACCATTACC	491 bp
CasMINI-1R	TTAAACAGTGCTGCTGCACGACGATAACAAACACGACTCAGATAATGTTCAACGCTGC	
CasMINI-2F	ATCTGAGTCGTGTTTGTTATCGTCGTGCAGCAGCACTGTTTAAAAACGCAGCAATTGC	1195 bp
CasMINI-2R	ACCTTGCGCTTCTTCTTGGGGCTAGCCGGTTCCTCTTTGGTGCTTTTC	
	ACCTTGCGCTTCTTCTTGGGGCTAGCCGGTTCCTCTTTGGTGCTTTTC	
CasMINI-F	TTTGCCGCCAGAACACAGCCTAGGGCCACCATGCCCAAGAAGAAGCGCAAG	1686 bp
CasMINI-R	AAGACTTCCTCTGCCCTCGACTTTCCTCTTCTTCTTTGGCGG	
2AEGFP-pA-F	AAAGAAGAAGAGGAAAGTCGAGGGCAGAGGAAGTCTTCTAAC	952 bp
2AEGFP-pA-R	AATCATGGGAAATAGGCCCTCGGTACCAATAAGATACATTGATGAGTTTGGACAAACC	
U6-ge4.1-Nr2e3-F	AAACTCATCAATGTATCTTATTGGTACCGAGGGCCTATTTCCCATGATTCC	440 bp
U6-ge4.1-Nr2e3-1R	ACTCCATCACTAGGGGTTCCTTCTAGAAAAAAAAAAATAAAAGGCGGTGATAAGGCTG	
U6-ge4.1-Nr2e3-4R	ACTCCATCACTAGGGGTTCCTTCTAGAAAAAAAAAAATAAAAATCACCGCCGAAACTTG	440 bp
pRK-F	TCCATCACTAGGGGTTCCTGCGCTCGAGGGGCCCCAGAAGCCTGGTG	343 bp
pRK-R	TTCTTGGGCATGGTGGCCCTAGGGCCCTTGGCCTGTGGCCC	

Table S4 Primers used for T7E1 assay and Sanger sequencing in this study.

Primer ID	Forward Primer	Reverse Primer	Product length
Nr2e3-1	GGAAAGCGGAAGACGAGA	CACAAGTACCCTAAGGTAAACTATG	885 bp
Nr2e3-2	CTCGGAGCATGGCTCAGGT	GGAGGTTTGGAGTTCAGTGGC	273 bp

Table S5 Barcode primers used for targeting efficiency analysis in this study.

Target ID	Forward Primer	Reverse Primer
Nr2e3-1	ATCACGCTCGGAGCATGGCTCAGGT	ATCACGGGAGGTTTGGAGTTCAGTGGC
	CGATGTCTCGGAGCATGGCTCAGGT	CGATGTGGAGGTTTGGAGTTCAGTGGC
	TTAGGCCTCGGAGCATGGCTCAGGT	TTAGGCGGAGGTTTGGAGTTCAGTGGC
	TGACCACTCGGAGCATGGCTCAGGT	TGACCAGGAGGTTTGGAGTTCAGTGGC
Nr2e3-4	ACAGTGCTCGGAGCATGGCTCAGGT	ACAGTGGGAGGTTTGGAGTTCAGTGGC
	GCCAATCTCGGAGCATGGCTCAGGT	GCCAATGGAGGTTTGGAGTTCAGTGGC
	CAGATCCTCGGAGCATGGCTCAGGT	CAGATCGGAGGTTTGGAGTTCAGTGGC
	ACTTGACTCGGAGCATGGCTCAGGT	ACTTGAGGAGGTTTGGAGTTCAGTGGC

Table S6 Primers used for targeted deep sequencing in this study.

Target ID	ON/OFF-Target	Forward Primer	Reverse Primer
Nr2e3-1	ON	GTCTCCCTGCTTTCCAAG	TGAGTATCCACGCCCAC
	OT1	CAAGCAGGCATGAACCAA	TCATCCAGACTTCAGGGAGAT
	OT2	TCAGGTTGGCAATGAGG	TGTTGGGTCCGACTTAT
	OT3	GGCTGTATTTCTGCTATGT	CGTGCAAGGGAGTTTA
	OT4	CTACCTGTAACACGGATTGA	CCCACTACCACCAACAAA
	OT5	GAATGAGCAGGGTTGT	GGGAAGCAGATTAGCG
	OT6	GCCGAGTCTGAACCAT	CCTTACTGTCCAGGTCTT
	OT7	TATAAAGAGGCAAGCAA	ATTCAAGGGTAAAGTGG
	OT8	GTGATCTAATGCCATGC	CTCTGTGAGACTTGCTTC
	OT9	GTGATTCGGGTTGATGATGG	CTGATTTGGGAGCGAGGG
	OT10	CAGGCTAATTTATGAACG	TTAGGAGGTTGGGAGAA
	OT11	GCTACTGAGCGTCTACTGC	GGTTTGGAGACAGGGTT
	OT12	TACCCATTGCCTTAACTA	GAGAAAATAGAGTGGAAACA
Nr2e3-4	ON	AGAGGAGGAGGTTTGGAGTT	GGAAACAGGCAGTGACCC
	OT1	AGAGGAAAGGGTCCCAGAC	ATGAGCGCCGAGCAAT
	OT2	CTCCCAGAAAATGAC	CACTGAAACCAAAATA
	OT3	AACTTTTGTGGATTTG	ATGCCTTAGGTATGAA
	OT4	CTCATACTCTTGGCTTCA	CAATTTCTCCTAGCACC
	OT5	CTCAGTTGCCTCTAGTTC	ACCCAGTGGCTACATC
	OT6	CCATTTGCCGCTGGAC	GAGGGAGTTTGCTATGATATTGTG
	OT7	TAAATGATTCAAAGCCC	AGACCAACCTATTCTAACA
	OT8	TCCCACTGCCCTAATG	GGAAGAGGTCGGAAGAA
	OT9	AGAAGGTCTGATAGGGAT	CTTTATGCAGGAATAATG
	OT10	CCTAAGTGGATATGCT	TCAGTTGTGATGTGGT
	OT11	GAATTAACAGGAGCATT	AGGGAACCTGGACTT
	OT12	ACATGGGCTAGGTGGAT	CAGAAGGCGAAGGAGTT
	OT13	TGGGCCAGTCTTCTGTTTCT	TTCGTCAGGCAGGTTCATC
	OT14	GAGGTCTGTAGCCTTCTGT	TTATGCCTTGCTTCTTGT
	OT15	CTGACGGGCAGGAAGTT	GGACAGAGGAGCAGAGGAG

Table S7 Antibodies used for immunofluorescence.

Antibody	Source	Species	Catalog No.	Dilution
RHO	Abcam	mouse	ab98887	1:500
S-opsin	Novus	rabbit	NBP1-20194	1:100
GFP	Abcam	chicken	ab13970	1:500
Mouse IgG	Invitrogen	donkey	A10036	1:1000
Chicken IgY	Invitrogen	goat	A11039	1:1000
Rabbit IgG	Invitrogen	goat	A21207	1:1000

Supplementary Sequences

Un1Cas12f1

ATGGCCAAAAACACCATTACCAAAACACTGAAACTGCGTATTGTGCGTCCGTATAATAGCGCAGAAGTGGAAAAAATTG
TTGCCGACGAAAAAAACAACCGCGAAAAAATCGCACTGGAAAAGAACAAAGACAAAGTGAAAGAAGCCTGCAGCAAACA
TCTGAAAGTTGCAGCATATTGTACCACACAGGTTGAACGTAATGCATGCCTGTTTTGTAAAGCACGTAAACTGGATGAC
AAATTCTACCAAAAACTGCGTGGTCAGTTTCCGGATGCAGTTTTTTGGCAAGAAATCAGCGAAATTTTTCGCCAGCTGC
AGAAACAGGCAGCAGAAATCTATAATCAGAGCCTGATCGAACTGTACTACGAGATTTTTATCAAAGGCAAAGGTATTGC
AAATGCCAGCAGCGTTGAACATTATCTGAGTGATGTTTGTTATACCCGTGCAGCAGAACTGTTTAAAAACGCAGCAATT
GCAAGCGGTCTGCGTAGCAAAATCAAAAGCAATTTTCGTCTGAAAGAACTGAAAAACATGAAAAGTGGTCTGCCGACCA
CCAAAAGCGATAATTTTCCGATTCCGCTGGTTAAACAGAAAGGTGGTCAGTATACCGGTTTTGAAATTAGCAATCATAA
TAGCGACTTCATCATCAAGATTCCGTTTGGTCGTTGGCAGGTCAAAAAAGAGATTGATAAATATCGTCCGTGGGAGAAA
TTTGACTTTGAACAGGTTCAGAAAAGCCCGAAACCGATTAGCCTGCTGCTGAGCACCCAGCGTCGTAAACGTAATAAAG
GTTGGAGCAAAGATGAAGGCACCGAAGCCGAAATCAAAAAAGTTATGAATGGCGATTATCAGACCAGCTACATTGAAGT
TAAACGTGGCAGCAAAATCGGTGAAAAAAGCGCATGGATGCTGAATCTGAGCATTGATGTTCCGAAAATTGATAAAGGT
GTGGATCCGAGCATTATTGGTGGTATTGATGTTGGTGTTAAATCACCGCTGGTTTGCGCAATTAACAATGCATTTAGCC
GTTATAGCATCAGCGATAACGACCTGTTTCACTTCAACAAGAAAATGTTTGCACGTCGTCGTATCCTGCTGAAAAAAAA
CCGTCATAAACGTGCAGGTCATGGTGCAAAAAACAAACTGAAACCGATCACCATTCTGACCGAAAAAAGTGAACGTTTT
CGCAAAAAGCTGATTGAACGTTGGGCATGTGAAATCGCGGATTTCTTCATTAAAAACAAAGTTGGCACCGTGCAGATGG
AAAATCTGGAAAGCATGAAACGTAAAGAGGACAGCTATTTTAACATTCGCCTGCGTGGCTTTTGGCCGTATGCAGAAAT
GCAGAACAAAATCGAATTCAAACTGAAGCAGTATGGCATCGAAATTCGTAAAGTTGCACCGAATAATACCAGCAAAACC
TGTAGCAAATGTGGCCATCTGAACAACTATTTCAACTTCGAGTACCGCAAGAAAAACAAATTCCCGCACTTTAAATGCG
AAAAATGCAACTTCAAAGAAAACGCCGATTATAATGCAGCCCTGAATATTTCAAACCCGAAACTGAAAAGCACCAAAGA
GGAACCG

CasMINI_v3.1

ATGGCCAAAAACACCATTACCAAAACACTGAAACTGCGTATTGTGCGTCCGTATAATAGCGCAGAAGTGGAAAAAATTG
TTGCCGACGAAAAAAACAACCGCGAAAAAATCGCACTGGAAAAGAACAAAGACAAAGTGAAAGAAGCCTGCAGCAAACA
TCTGAAAGTTGCAGCATATTGTACCACACAGGTTGAACGTAATGCATGCCTGTTTTGTAAAGCACGTAAACTGGATGAC
AAATTCTACCAAAAACTGCGTGGTCAGTTTCCGGATGCAGTTTTTTGGCAAGAAATCAGCGAAATTTTTCGCCAGCTGC
AGAAACAGGCAGCAGAAATCTATAATCAGAGCCTGATCGAACTGTACTACGAGATTTTTATCAAAGGCAAAGGTATTGC
AAATGCCAGCAGCGTTGAACATTATCTGAGTCGTGTTTGTTATCGTCGTGCAGCAGCACTGTTTAAAAACGCAGCAATT
GCAAGCGGTCTGCGTAGCAAAATCAAAAGCAATTTTCGTCTGAAAGAACTGAAAAACATGAAAAGTGGTCTGCCGACCA
CCAAAAGCGATAATTTTCCGATTCCGCTGGTTAAACAGAAAGGTGGTCAGTATACCGGTTTTGAAATTAGCAATCATAA
TAGCGACTTCATCATCAAGATTCCGTTTGGTCGTTGGCAGGTCAAAAAAGAGATTGATAAATATCGTCCGTGGGAGAAA
TTTGACTTTGAACAGGTTCAGAAAAGCCCGAAACCGATTAGCCTGCTGCTGAGCACCCAGCGTCGTAAACGTAATAAAG
GTTGGAGCAAAGATGAAGGCACCGAAGCCGAAATCAAAAAAGTTATGAATGGCGATTATCAGACCAGCTACATTGAAGT
TAAACGTGGCAGCAAAATCGGTGAAAAAAGCGCATGGATGCTGAATCTGAGCATTGATGTTCCGAAAATTGATAAAGGT
GTGGATCCGAGCATTATTGGTGGTATTGATGTTGGTGTTAAATCACCGCTGGTTTGCGCAATTAACAATGCATTTAGCC
GTTATAGCATCAGCGATAACGACCTGTTTCACTTCAACAAGAAAATGTTTGCACGTCGTCGTATCCTGCTGAAAAAAAA
CCGTCATAAACGTGCAGGTCATGGTGCAAAAAACAAACTGAAACCGATCACCATTCTGACCGAAAAAAGTGAACGTTTT
CGCAAAAAGCTGATTGAACGTTGGGCATGTGAAATCGCGGATTTCTTCATTAAAAACAAAGTTGGCACCGTGCAGATGG
AAAATCTGGAAAGCATGAAACGTAAAGAGGACAGCTATTTTAACATTCGCCTGCGTGGCTTTTGGCCGTATGCAGAAAT
GCAGAACAAAATCGAATTCAAACTGAAGCAGTATGGCATCGAAATTCGTAAAGTTGCACCGAATAATACCAGCAAAACC

TGTAGCAAATGTGGCCATCTGAACAACTATTTCAACTTCGAGTACCGCAAGAAAAACAAATTCCCGCACTTTAAATGCG
AAAAATGCAACTTCAAAGAAAACGCCGATTATAATGCAGCCCTGAATATTTCAAACCCGAAACTGAAAAGCACCAAAGA
GGAACCG

AsCas12f1

ATGATCAAAGTATATCGGTATGAGATCGTAAAACCGCTTGATTTAGATTGGAAAGAGTTTGGAACTATTTTACGACAGT
TACAACAGGAAACTCGTTTTGCCTTAAACAAAGCGACTCAACTGGCTTGGGAATGGATGGGCTTTAGTAGTGATTATAA
GGATAATCATGGGGAATATCCAAAAAGTAAAGACATTCTCGGATACACGAATGTTCACGGATACGCTTACCATACGATC
AAAACCAAAGCATATCGCTTAAACAGTGGTAATTTATCTCAAACCATCAAACGTGCGACGGATCGTTTTAAAGCATATC
AAAAGGAAATCTTACGTGGCGATATGTCCATTCCGTCCTATAAACGAGATATTCCACTCGATCTGATTAAAGAGAATAT
CAGCGTAAATCGTATGAATCACGGGGATTATATCGCTTCGTTATCTCTGTTAAGCAATCCAGCCAAGCAAGAAATGAAT
GTAAAGAGGAAAATTTCGGTCATAATCATTGTCCGTGGCGCAGGGAAAACAATCATGGACAGAATCCTAAGTGGTGAAT
ACCAAGTCTCTGCTTCACAAATAATTCACGATGATCGAAAAAACAAATGGTATTTAAACATAAGTTATGATTTTGAACC
CCAAACCCGAGTGTTGGATCTAAATAAAATAATGGGGATTGATTTAGGCGTTGCTGTTGCCGTTTACATGGCATTTCAA
CATACCCCTGCTCGATACAAATTGGAGGGAGGTGAGATTGAAAATTTTCGTAGACAGGTAGAATCTCGACGGATTAGTA
TGTTACGTCAAGGTAAATATGCCGGTGGTGCCCGTGGAGGGCATGGGCGAGATAAAAGAATAAAACCGATTGAACAGCT
TCGAGATAAAATTGCTAATTTTAGAGATACCACAAACCATCGTTATAGTAGATATATAGTGGATATGGCAATTAAAGAA
GGTTGTGGAACGATTCAGATGGAGGATTTAACGAATATACGCGATATTGGATCTCGATTTTTACAAAATTGGACCTATT
ATGATTTGCAGCAAAAAATTATTTATAAAGCCGAAGAGGCAGGAATTAAAGTCATAAAGATCGATCCCCAATATACCAG
TCAACGTTGTAGTGAATGTGGTAATATAGATTCCGGGAATCGAATTGGACAAGCTATCTTTAAATGCAGAGCTTGCGGA
TACGAAGCCAATGCAGACTACAATGCTGCACGAAACATAGCAATACCGAACATTGACAAAATTATAGCTGAGAGTATCA
AA

ge4.1

ACCGCTTCACTTAGAGTGAAGGTGGGCTGCTTGCATCAGCCTAATGTCGAGAAGTGCTTTCTTCGGAAAGAACCCTCGA
AACAAAGAAAGGAATGCAACNNNNNNNNNNNNNNNNNNNNTTTTATTTT

ge4.0

ACCGCTTCACCAAAAGCTGTCCCTTAGGGGATTAGAACTTGAGTGAAGGTGGGCTGCTTGCATCAGCCTAATGTCGAGA
AGTGCTTTCTTCGGAAAGAACCCTCGAAACAAAGAAAGGAATGCAACNNNNNNNNNNNNNNNNNNNNTTTTATTTT

Design2

GGGCTTCACTGATAAAGTGGAGAACCGCTTCACCAAAAGCTGTCCCTTAGGGGATTAGAACTTGAGTGAAGGTGGGCTG
CTTGCATCAGCCTAATGTCGAGAAGTGCTTTCTTCGGAAAGTAACCCTCGAAACAAATTCATTTGAATGAAGGAATGCA
ACNNNNNNNNNNNNNNNNNNNN

AsgRNA

ATTCGTCGGTTCAGCGACGATAAGCCGAGAAGTGCCAATAAAACTGTTAAGTGGTTTGGTAACGCTCGGTAAGGTAGCC
AAAAGGCTGAAACTCCGTGCACAAAGACCGCACGGACGCTTCACATATAGCTCATAAACAAAGTTTGCGAGCTAGCTTG
TGGAGTGTGAACNNNNNNNNNNNNNNNNNNNN

AAV-CasMINI_v3.1-ge4.1-*Nr2e3*

ITR-pRK-CasMINI_v3.1-2A-EGFP-pA-U6-ge4.1-*Nr2e3*-ITR

CCTGCAGGCAGCTGCGCGCTCGCTCGCTCACTGAGGCCGCCCGGGCGTCGGGCGACCTTTGGTCGCCCGGCCTCAGTGA

GCGAGCGAGCGCGCAGAGAGGGAGTGGCCAACTCCATCACTAGGGGTTCCTGCGCTCGAGGGGCCCCAGAAGCCTGGTG
GTTGTTTGTCCTTCTCAGGGGAAAAGTGAGGCGGCCCCTTGGAGGAAGGGGCCGGGCAGAATGATCTAATCGGATTCCA
AGCAGCTCAGGGGATTGTCTTTTTCTAGCACCTTCTTGCCACTCCTAAGCGTCCTCCGTGACCCCGGCTGGGATTTAGC
CTGGTGCTGTGTCAGCCCCGGTCTCCCAGGGGCTTCCCAGTGGTCCCCAGGAACCCTCGACAGGGCCCGGTCTCTCTCG
TCCAGCAAGGGCAGGGACGGGCCACAGGCCAAGGGCCCTAGGGCCACCATGCCCAAGAAGAAGCGCAAGGTGCCCGGGA
TGGCCAAAAACACCATTACCAAAACACTGAAACTGCGTATTGTGCGTCCGTATAATAGCGCAGAAGTGGAAAAAATTGT
TGCCGACGAAAAAAACAACCGCGAAAAAATCGCACTGGAAAAGAACAAAGACAAAGTGAAAGAAGCCTGCAGCAAACAT
CTGAAAGTTGCAGCATATTGTACCACACAGGTTGAACGTAATGCATGCCTGTTTTGTAAAGCACGTAAACTGGATGACA
AATTCTACCAAAAACTGCGTGGTCAGTTTCCGGATGCAGTTTTTTGGCAAGAAATCAGCGAAATTTTTCGCCAGCTGCA
GAAACAGGCAGCAGAAATCTATAATCAGAGCCTGATCGAACTGTACTACGAGATTTTTATCAAAGGCAAAGGTATTGCA
AATGCCAGCAGCGTTGAACATTATCTGAGTCGTGTTTGTTATCGTCGTGCAGCAGCACTGTTTAAAAACGCAGCAATTG
CAAGCGGTCTGCGTAGCAAAATCAAAAGCAATTTTCGTCTGAAAGAACTGAAAAACATGAAAAGTGGTCTGCCGACCAC
CAAAAGCGATAATTTTCCGATTCCGCTGGTTAAACAGAAAGGTGGTCAGTATACCGGTTTTGAAATTAGCAATCATAAT
AGCGACTTCATCATCAAGATTCCGTTTGGTCGTTGGCAGGTCAAAAAAGAGATTGATAAATATCGTCCGTGGGAGAAAT
TTGACTTTGAACAGGTTCAGAAAAGCCCGAAACCGATTAGCCTGCTGCTGAGCACCCAGCGTCGTAAACGTAATAAAGG
TTGGAGCAAAGATGAAGGCACCGAAGCCGAAATCAAAAAAGTTATGAATGGCGATTATCAGACCAGCTACATTGAAGTT
AAACGTGGCAGCAAAATCGGTGAAAAAAGCGCATGGATGCTGAATCTGAGCATTGATGTTCCGAAAATTGATAAAGGTG
TGGATCCGAGCATTATTGGTGGTATTGATGTTGGTGTTAAATCACCGCTGGTTTGCGCAATTAACAATGCATTTAGCCG
TTATAGCATCAGCGATAACGACCTGTTTCACTTCAACAAGAAAATGTTTGCACGTCGTCGTATCCTGCTGAAAAAAAAC
CGTCATAAACGTGCAGGTCATGGTGCAAAAAACAAACTGAAACCGATCACCATTCTGACCGAAAAAAGTGAACGTTTTC
GCAAAAAGCTGATTGAACGTTGGGCATGTGAAATCGCGGATTTCTTCATTAAAAACAAAGTTGGCACCGTGCAGATGGA
AAATCTGGAAAGCATGAAACGTAAAGAGGACAGCTATTTTAACATTCGCCTGCGTGGCTTTTGGCCGTATGCAGAAATG
CAGAACAAAATCGAATTCAAACTGAAGCAGTATGGCATCGAAATTCGTAAAGTTGCACCGAATAATACCAGCAAAACCT
GTAGCAAATGTGGCCATCTGAACAACTATTTCAACTTCGAGTACCGCAAGAAAAACAAATTCCCGCACTTTAAATGCGA
AAAATGCAACTTCAAAGAAAACGCCGATTATAATGCAGCCCTGAATATTTCAAACCCGAAACTGAAAAGCACCAAAGAG
GAACCGCCTAAGAAAAAAAGGAAGGTGACTAGTGAGGGCAGAGGAAGTCTTCTAACATGCGGTGACGTGGAGGAGAATC
CCGGCCCAATGGTGAGCAAGGGCGAGGAGCTGTTCACCGGGGTGGTGCCCATCCTGGTCGAGCTGGACGGCGACGTAAA
CGGCCACAAGTTCAGCGTGTCCGGCGAGGGCGAGGGCGATGCCACCTACGGCAAGCTGACCCTGAAGTTCATCTGCACC
ACCGGCAAGCTGCCCGTGCCCTGGCCCACCCTCGTGACCACCCTGACCTACGGCGTGCAGTGCTTCAGCCGCTACCCCG
ACCACATGAAGCAGCACGACTTCTTCAAGTCCGCCATGCCCGAAGGCTACGTCCAGGAGCGCACCATCTTCTTCAAGGA
CGACGGCAACTACAAGACCCGCGCCGAGGTGAAGTTCGAGGGCGACACCCTGGTGAACCGCATCGAGCTGAAGGGCATC
GACTTCAAGGAGGACGGCAACATCCTGGGGCACAAGCTGGAGTACAACTACAACAGCCACAACGTCTATATCATGGCCG
ACAAGCAGAAGAACGGCATCAAGGTGAACTTCAAGATCCGCCACAACATCGAGGACGGCAGCGTGCAGCTCGCCGACCA
CTACCAGCAGAACACCCCCATCGGCGACGGCCCCGTGCTGCTGCCCGACAACCACTACCTGAGCACCCAGTCCGCCCTG
AGCAAAGACCCCAACGAGAAGCGCGATCACATGGTCCTGCTGGAGTTCGTGACCGCCGCCGGGATCACTCTCGGCATGG
ACGAGCTGTACAAGTAAGCTAGCCCAACTTGTTTATTGCAGCTTATAATGGTTACAAATAAAGCAATAGCATCACAAAT
TTCACAAATAAAGCATTTTTTTCACTGCATTCTAGTTGTGGTTTGTCCAAACTCATCAATGTATCTTATTGGTACCGAG
GGCCTATTTCCCATGATTCCTTCATATTTGCATATACGATACAAGGCTGTTAGAGAGATAATTGGAATTAATTTGACTG
TAAACACAAAGATATTAGTACAAAATACGTGACGTAGAAAGTAATAATTTCTTGGGTAGTTTGCAGTTTTAAAATTATG
TTTTAAAATGGACTATCATATGCTTACCGTAACTTGAAAGTATTTCGATTTCTTGGCTTTATATATCTTGTGGAAAGGA
CGAAACACCGGACCGCTTCACTTAGAGTGAAGGTGGGCTGCTTGCATCAGCCTAATGTCGAGAAGTGCTTTCTTCGGAA
AGAACCCTCGAAACAAAGAAAGGAATGCAAC*TGGCCAGCCTTATCACCGCC*TTTTATTTTTTTTTTTCTAGAAGGAACC
CCTAGTGATGGAGTTGGCCACTCCCTCTCTGCGCGCTCGCTCGCTCACTGAGGCCGGGCGACCAAAGGTCGCCCGACGC
CCGGGCTTTGCCCGGGCGGCCTCAGTGAGCGAGCGAGCGCGCAGCTGCCTGCAGG

pAAV-EF1α backbone

ITR-EF1α-*AvrII*-*XbaI*-ITR

AAAAATAAACAAATAGGGGTTCCGCGCACATTTCCCCGAAAAGTGCCACCTGACGTCTAAGAAACCATTATTATCATGA
CATTAACCTATAAAAATAGGCGTATCACGAGGCCCTTTCGTTGTAAAACGACGGCCAGTCGGTCTCGATCCGCAGTGTC
TTGGGTCTCTCCTGCAGGCAGCTGCGCGCTCGCTCGCTCACTGAGGCCGCCCGGGCGTCGGGCGACCTTTGGTCGCCCG
GCCTCAGTGAGCGAGCGAGCGCGCAGAGAGGGAGTGGCCAACTCCATCACTAGGGGTTCCTGCGCTCGAGGGGCAGAGC
GCACATCGCCCACAGTCCCCGAGAAGTTGGGGGGAGGGGTCGGCAATTGATCCGGTGCCTAGAGAAGGTGGCGCGGGGT
AAACTGGGAAAGTGATGTCGTGTACTGGCTCCGCCTTTTTCCCGAGGGTGGGGGAGAACCGTATATAAGTGCAGTAGTC
GCCGTGAACGTTCTTTTTCGCAACGGGTTTGCCGCCAGAACACAG*CCTAGGTCTAGA*AGGAACCCCTAGTGATGGAGTT
GGCCACTCCCTCTCTGCGCGCTCGCTCGCTCACTGAGGCCGGGCGACCAAAGGTCGCCCGACGCCCGGGCTTTGCCCGG
GCGGCCTCAGTGAGCGAGCGAGCGCGCAGCTGCCTGCAGGAGAGACCGAGTCACTGCCAACCGAGACCGGTCATAGCTG
TTTCCTGTGTGCCGCTTCCTCGCTCACTGACTCGCTGCGCTCGGTCGTTCGGCTGCGGCGAGCGGTATCAGCTCACTCA
AAGGCGGTAATACGGTTACCCACAGAATCAGGGGATAACGCAGGAAAGAACATGTGAGCAAAAGGCCAGCAAAAGGCCA
GGAACCGTAAAAAGGCCGCGTTGCTGGCGTTTTTCCATAGGCTCCGCCCCCCTGACGAGCATCACAAAAATCGACGCTC
AAGTCAGAGGTGGCGAAACCCGACAGGACTATAAAGATACCAGGCGTTTCCCCCTGGAAGCTCCCTCGTGCGCTCTCCT
GTTCCGACCCTGCCGCTTACCGGATACCTGTCCGCCTTTCTCCCTTCGGGAAGCGTGGCGCTTTCTCATAGCTCACGCT
GTAGGTATCTCAGTTCGGTGTAGGTCGTTCGCTCCAAGCTGGGCTGTGTGCACGAACCCCCCGTTCAGCCCGACCGCTG
CGCCTTATCCGGTAACTATCGTCTTGAGTCCAACCCGGTAAGACACGACTTATCGCCACTGGCAGCAGCCACTGGTAAC
AGGATTAGCAGAGCGAGGTATGTAGGCGGTGCTACAGAGTTCTTGAAGTGGTGGCCTAACTACGGCTACACTAGAAGGA
CAGTATTTGGTATCTGCGCTCTGCTGAAGCCAGTTACCTTCGGAAAAAGAGTTGGTAGCTCTTGATCCGGCAAACAAAC
CACCGCTGGTAGCGGTGGTTTTTTTGTTTGCAAGCAGCAGATTACGCGCAGAAAAAAAGGATCTCAAGAAGATCCTTTG
ATCTTTTCTACGGGGTCTGACGCTCAGTGGAACGAAAACTCACGTTAAGGGATTTTGGTCATGAGATTATCAAAAAGGA
TCTTCACCTAGATCCTTTTAAATTAAAAATGAAGTTTTAAATCAATCTAAAGTATATATGAGTAAACTTGGTCTGACAG
TTAGAAAAACTCATCGAGCATCAAATGAAACTGCAATTTATTCATATCAGGATTATCAATACCATATTTTTGAAAAAGC
CGTTTCTGTAATGAAGGAGAAAACTCACCGAGGCAGTTCCATAGGATGGCAAGATCCTGGTATCGGTCTGCGATTCCGA
CTCGTCCAACATCAATACAACCTATTAATTTCCCCTCGTCAAAAATAAGGTTATCAAGTGAGAAATCACCATGAGTGAC
GACTGAATCCGGTGAGAATGGCAAAAGTTTATGCATTTCTTTCCAGACTTGTTCAACAGGCCAGCCATTACGCTCGTCA
TCAAAATCACTCGCATCAACCAAACCGTTATTCATTCGTGATTGCGCCTGAGCGAGTCGAAATACGCGATCGCTGTTAA
AAGGACAATTACAAACAGGAATCGAATGCAACCGGCGCAGGAACACGGCCAGCGCATCAACAATATTTTCACCTGAATC
AGGATATTCTTCTAATACCTGGAATGCTGTTTTCCCGGGGATCGCTGTGGTGAGTAACCATGCATCATCAGGAGTACGG
ATAAAATGCTTGATGGTCGGAAGAGGCATAAATTCCGTCAGCCAGTTTAGTCTGACCATCTCATCTGTAACATCATTGG
CAACGCTACCTTTGCCATGTTTCAGAAACAACTCTGGCGCATCGGGCTTCCCATACAATCGATAGATTGTCGCACCTGA
TTGCCCGACATTATCGCGAGCCCATTTATACCCATATAAATCAGCATCCATGTTGGAATTTAATCGCGGCCTAGAGCAA
GACGTTTCCCGTTGAATATGGCTCATACTCTTCCTTTTTCAATATTATTGAAGCATTTATCAGGGTTATTGTCTCATGA
GCGGATACATATTTGAATGTATTTAG
